# Effective Anti-tumor Response by TIGIT Blockade Associated With FcγR Engagement and Myeloid Cell Activation

**DOI:** 10.3389/fimmu.2020.573405

**Published:** 2020-10-07

**Authors:** Jin-Hwan Han, Mingmei Cai, Jeffery Grein, Samanthi Perera, Hongmei Wang, Mike Bigler, Roenna Ueda, Thomas W. Rosahl, Elaine Pinheiro, Drake LaFace, Wolfgang Seghezzi, Sybil M. Genther Williams

**Affiliations:** Merck & Co. Inc., Kenilworth, NJ, United States

**Keywords:** TIGIT, FcγR, myeloid cells, combination cancer immunotherapy, costimulatory molecules, immune checkpoint blockade

## Abstract

The molecule “T cell immunoreceptor with immunoglobulin and ITIM domain,” or TIGIT, has recently received much attention as a promising target in the treatment of various malignancies. In spite of the quick progression of anti-TIGIT antibodies into clinical testing both as monotherapy and in combination with programmed cell death-1 (PD-1)–directed immune checkpoint blockade, the molecular mechanism behind the observed therapeutic benefits remains poorly understood. Here we demonstrate, using mouse tumor models, that TIGIT blocking antibodies with functional Fc binding potential induce effective anti-tumor response. Our observations reveal that the anti-TIGIT therapeutic effect is not achieved by depletion of intratumoral regulatory T cells (Treg) or any cell population expressing TIGIT, but instead is mediated by possible “reverse activating signals” through FcγRs on myeloid cells, inducing expression of various mediators such as cytokines and chemokines. Furthermore, we discovered an induction of a robust and persistent granzyme B and perforin response, distinct from a predominantly interferon-γ (IFN-γ)-driven anti-PD-1 blockade. Our observations, for the first time, provide the basis for a mechanistic hypothesis involving the requirement of a functional Fc domain of anti-TIGIT monoclonal antibodies, of which various isotypes are currently under intense clinical investigation.

## Introduction

Immunotherapy has been revolutionizing the treatment of patients with cancer by opening a new paradigm for anti-cancer therapy that has traditionally relied on use of cytotoxics, surgery or cancer cell targeted therapies. While the concept of harnessing the immune system to recognize and fight cancerous cells with somatic mutations has existed since the 19th century, it was only in the past few years that we have witnessed the successful translation of this concept into the clinical setting. So far, the most promising and broadly applied approach involves the use of antibodies blocking co-inhibitory receptors on cells of the immune system that typically harbor an intracellular immunoreceptor tyrosine-based inhibitory motif (ITIM), such as PD-1 or cytotoxic T-lymphocyte-associated protein 4 (CTLA-4). While immunotherapy has significantly improved cancer care in specific cancer types and segments of patient populations, the majority of patients still does not respond to these therapies, which has fueled intense research to identify additional molecular targets with possibly distinct or complementary pathways offering promise for successful therapeutic interference in mono- or combination therapy settings. One such promising target is *T* cell immunoreceptor with *i*mmuno*g*lobulin and *IT*IM domain, or TIGIT, identified in 2009 by a genomic search from activated T cells (1). TIGIT has been shown to be up-regulated on activated CD4 T cells, including Tregs, natural killer (NK) cells (1, 2), tumor antigen-specific CD8^+^ T cells and on NK cells where, when engaged, TIGIT directly inhibits cytotoxicity (3). The known ligands for TIGIT are CD155 [also known as poliovirus receptor (PVR)] and CD112 (also known as nectin-2) that are expressed on myeloid, endothelial or tumor cells (1). A co-activating receptor on T cells, CD226, also called DNAX accessary molecule-1 (DNAM-1) (4), can also bind the same ligands but with about 100-folds lower binding affinity than TIGIT, having the opposite effect by enhancing cytotoxicity of T cells and NK cells. When TIGIT is up-regulated on antigen-specific T cells upon their activation, it will successfully outcompete CD226 to engage ligands leading to an immunosuppressive state (1). The same paradigm holds true for CTLA-4 where CTLA-4 has a higher affinity for CD80 and CD86 as compared to its co-activating receptor CD28. In preclinical models, anti-TIGIT antibodies have been shown to be efficacious in driving anti-tumor efficacy (5–7).

Here we have explored the role and requirement for IgG-Fc:FcγR interactions in addition to antagonism of TIGIT:CD155 binding in preclinical tumor models using anti-TIGIT antibodies as therapeutic agents. Unlike previous reports of antibodies against other immunomodulatory targets that require FcγR engagement in preclinical models [anti-CTLA-4, anti-glucocorticoid-induced tumor necrosis factor receptor (GITR)], Fc engagement of anti-TIGIT antibodies with FcγRs does not appear to induce depletion of cell populations that express TIGIT. Rather, we demonstrate that Fc engagement of anti-TIGIT antibodies induces a persistent immune activation through engagement of FcγR on myeloid cells leading to cytokine and chemokine production, and an enhanced antigen presentation, throughout the course of the therapeutic treatment. Additionally, we demonstrate that persistent TIGIT antagonism stimulates a robust induction of perforin and granzyme B release, both mechanisms of which are distinct from the situation observed in anti-PD-1, anti-CTLA-4 or anti-GITR treatments.

## Results

### An Anti-mTIGIT Antibody on a Mouse IgG2a Isotype Induces Anti-tumor Responses as a Monotherapy and in a Combination With an Anti-PD-1 Antibody

Multiple reports have demonstrated an immunomodulatory role for TIGIT, but only recently a role for TIGIT in tumor biology has been described in combination with anti-PD-1/L1 (5, 6). To elucidate the mechanism of action of antagonist TIGIT antibodies in anti-tumor responses, we generated a series of rat anti-mouse TIGIT antibodies based on *in vitro* binding and blocking assays [mTIGIT:mouse CD155 (mCD155) interaction] (Supplementary Figure 1A). While additional clones with similar *in vitro* and *in vivo* activities have been identified, experiments described in this manuscript utilize clone 18G10 which we have demonstrated to be representative for anti-mTIGIT blocking antibodies. In order to understand the potential effect of isotype on antibody function and anti-tumor activity, we also generated chimeric versions by replacing the Fc portion of the rat anti-mTIGIT antibodies with mouse IgG isotypes. We constructed antibody chimeras on either an intact mouse IgG2a (mIgG2a) isotype that is capable of binding to Fcγ receptors (FcγRs), or a mutant mouse IgG1 with a point mutation at position 265 aspartic acid (D) to alanine (A) in order to abrogate the interaction of the Fc portion of the antibody with FcγRs (mIgG1-[D265A], hereafter mIgG1^*^). We confirmed that the binding affinities to mTIGIT recombinant protein remained comparable regardless of the two different isotypes (Supplementary Figure 1B).

In order to understand how antibody isotype differences may impact the anti-tumor efficacy of anti-TIGIT antibodies we evaluated the antibodies as single agents and in combination with the anti-mouse PD-1 antibody, clone DX400 in mIgG1^*^ isotype (hereafter, anti-PD-1), in multiple mouse syngeneic tumor models. As it has been well-established that anti-PD-1 antibody induces an improved anti-tumor response with no functional Fc (8), we exclusively used an isotype (mIgG1^*^) that does not bind to FcγRs.

**Figure 1:**
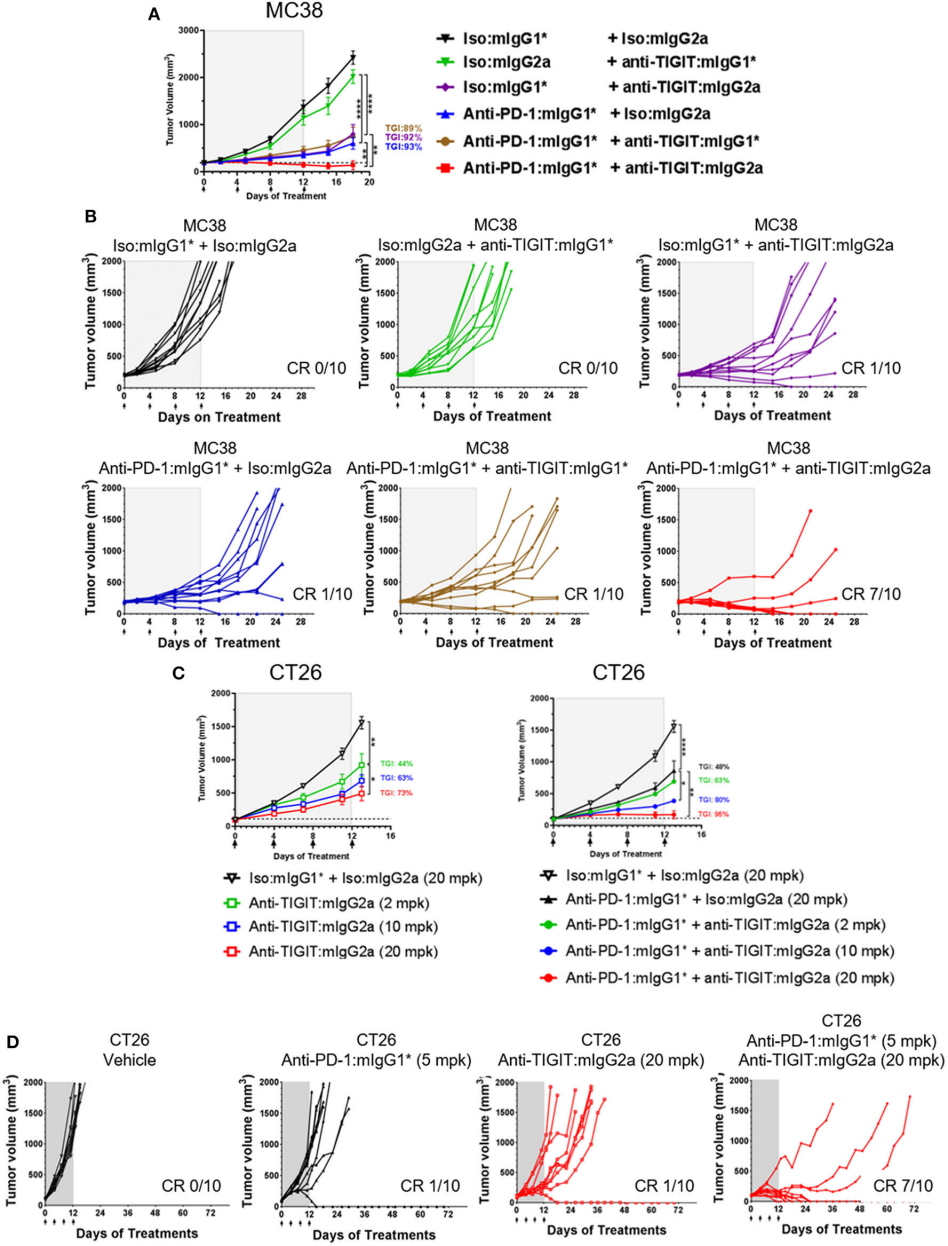
Anti-TIGIT antibody induces anti-tumor response with a certain isotype. **(A,B)** Large (average 190 mm^3^), established MC38-bearing mice were enrolled in each group (n=10 per group) and injected with antibodies i.p. every 4 days for times as indicated. **(C,D)** Antibody treatments for *in vivo* titration of anti-TIGIT:mIgG2a (2, 10, or 20 mpk) as a monotherapy or as a combination with anti-PD-1 (5 mpk) have been initiated when CT26 tumor was formed at average 98 mm^3^ subcutaneously (*n* = 10 per group). Dotted lines in **(A,C)** indicate the average tumor volumes at which the antibody treatments had been initiated. Data are representative of at least 3 independent experiments. *p < 0.05; **p < 0.01; ****p < 0.001.

The MC38 model is highly responsive to anti-PD-1 treatment with complete and durable regressions observed when treatment is started at small tumor volumes (~100 mm^3^). However, with larger starting tumor volumes (~190 mm^3^), only partial regressions are observed which allows for the evaluation of enhanced anti-tumor activity in an anti-TIGIT and anti-PD-1 combination approach. MC38 model selection was based on the presence of baseline TIGIT protein surface expression on CD8^+^ and CD4^+^ tumor infiltrating lymphocytes (Supplementary Figure 2A). A shown in Figure 1, we began treatment of MC38 tumor-bearing mice with anti-TIGIT: mIgG2a and anti-TIGIT:mIgG1^*^ as single agents, or in combination with anti-PD-1 when the tumor size was on average 190 mm^3^ (Figures 1A,B). Although the anti-TIGIT on both isotypes demonstrated equal binding to mTIGIT and blocking of mCD155, significant *in vivo* anti-tumor efficacy was observed only with the anti-TIGIT: mIgG2a antibody and not the anti-TIGIT:mIgG1^*^ antibody (Figures 1A,B). The anti-TIGIT:mIgG2a antibody showed a tumor growth inhibition (TGI) rate comparable to that of anti-PD-1 with a 92% and 93% TGI observed, respectively, with 10% (1/10) complete responses (CR) in both single agent groups (Figures 1A,B). The treatment of the MC38-bearing mice with the anti-TIGIT: mIgG1^*^ antibody as a single agent, on the other hand, showed little anti-tumor responses *in vivo* (Figure 1A, upper middle panel). Additionally, treatment of anti-TIGIT: mIgG2a with anti-PD-1 was significantly more efficacious than combination treatment with anti-TIGIT:mIgG1^*^. TGI for the anti-TIGIT: mIgG2a + anti-PD-1 combination was calculated as 100% with 7 out 10 mice undergoing complete responses to the combination therapy (Figures 1A,B). Serum was taken from a parallel pharmacokinetics (PK) cohort of mice during the dosing period to measure circulating anti-TIGIT: mIgG1^*^, anti-TIGIT: mIgG2a and anti-PD-1 drug levels to ensure the differences of *in vivo* efficacy were not due to different concentration of each antibody in the circulation. The drug exposure profiles (as well as binding affinities; Supplementary Figure 1B) of anti-TIGIT: mIgG2a and anti-TIGIT:mIgG1^*^ were found to be comparable (Supplementary Figure 3), suggesting that the differences of anti-tumor efficacy by anti-TIGIT antibodies with different isotypes can be attributed to differences in their biological activities.

To evaluate the ability of anti-TIGIT: mIgG2a to induce tumor growth inhibition either as mono- or combination therapy with anti-PD-1, the CT26 syngeneic tumor model was utilized. CT26 was chosen because it is less responsive to anti-PD-1 than MC38 (9), thus offers an opportunity to evaluate combination therapy effects between anti-PD-1 and anti-TIGIT. The expression of PD-1 and TIGIT on CD8 T cells and Tregs was also verified by flow cytometry analysis (Supplementary Figure 2B). CT26 syngeneic tumor model animals were treated with three increasing doses of anti-TIGIT:mIgG2a antibodies, yielding a clear dose dependence in tumor growth inhibition as demonstrated in Figures 1C,D (44, 63, and 73% TGI at 2, 10, 20 mpk, respectively). As in the fixed dose experiment in both MC38 and CT26 tumor models conducted earlier (Figures 1A,B), the combination of anti-PD-1 and anti-TIGIT: mIgG2a resulted in a significantly more prominent tumor response than anti-TIGIT:mIgG2a monotherapy. With the combination of 20 mpk of anti-TIGIT: mIgG2a with 10 mpk of anti-PD-1 in this model, 7 out of 10 complete regressions were observed (Figure 1D). Taken together, these data suggest a pronounced isotype advantage of the mIgG2a for the induction of anti-tumor response with anti-TIGIT antibodies.

### Blocking FcγRIV Significantly Reduces Efficacy of an Anti-TIGIT:mIgG2a Antibody

**Figure 2:**
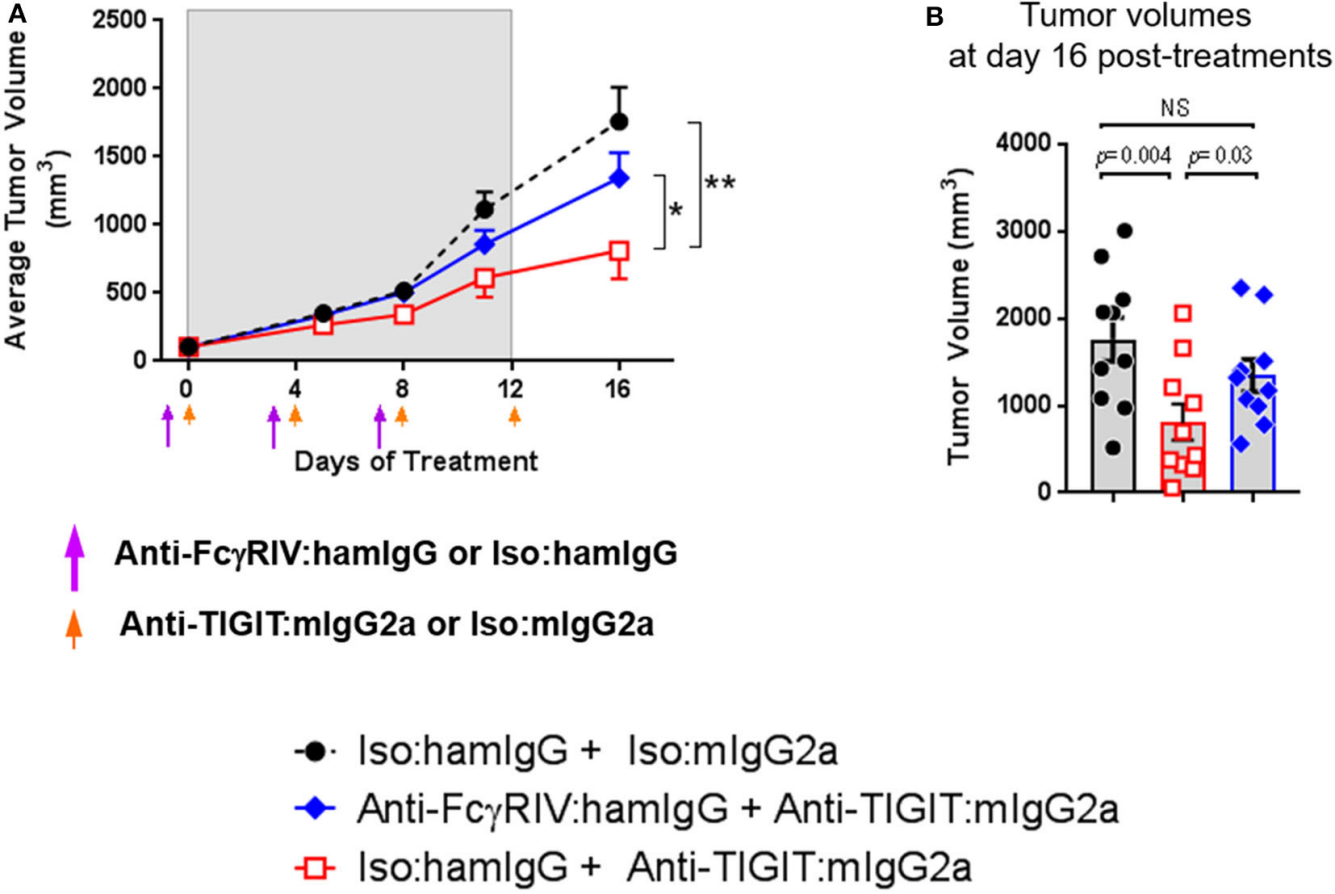
Anti-TIGIT antibody requires interaction with a specific FcγR. Tumor volume in the presence or absence of functional FcγRIV *in vivo* blocking 1 day prior to anti-TIGIT:mIgG2a was measured. **(A)** Changes of tumor volume and **(B)** tumor volume on individual mouse 16 days of the treatments are depicted. Anti-FcγRIV-specific antibody, 9E9, or its isotype control antibody (Armenian Hamster IgG) both at 10 mpk, was injected every 4 days i.p. (shown as purple arrow heads), 1 day before anti-TIGIT:mIgG2a or its isotype control antibody injection (shown as orange arrow heads) both at 18 mpk, to CT26-bearing mice in order to block functionally block FcγRIV *in vivo*. Ten mice were included in each group. Data are representative of 2 independent experiments. *p < 0.05; **p < 0.01.

The data demonstrating significantly enhanced anti-tumor efficacy with anti-TIGIT: mIgG2a antibody as compared to anti-TIGIT:mIgG1^*^ antibody suggests that the interaction of the Fc portion of the anti-TIGIT antibody with FcγRs plays a critical role in its ability to achieve anti-tumor activity. The specific receptor for mIgG2a isotype antibodies is FcγRIV that is expressed on myeloid cells, including neutrophils, monocytes, and macrophages (10), and we found that FcγRIV-expressing myeloid (CD11b^+^) cells are present not only in the spleen but also in the tumor (Supplementary Figure 4). While an experimental depletion of myeloid cells to understand their absolute mechanistic requirement in an anti-TIGIT response is fraught with challenges to interpret the outcome as it has been shown that the depletion of myeloid cells, which will include myeloid-derived suppressor cells or MDSCs, itself induces anti-tumor response (11). Instead, in order to experimentally address the requirement of the anti-TIGIT:mIgG2a antibody interaction with FcγRs in tumor growth inhibition, an antibody-mediated functional blockage of FcγRIV, the main receptor reported to bind the mIgG2a isotype (10), followed by anti-TIGIT: mIgG2a treatment was performed. This experimental approach was validated in previous work (12). We reason that the effect of anti-TIGIT: mIgG2a and FcγRIV can be investigated in the context of anti-tumor response without perturbing the cellular composition of TME with such an approach. Prior to performing the efficacy experiment we confirmed the blocking activity of the 9E9 antibody and tested duration of 9E9 binding to FcγRIV *in vivo* to inform on an ideal dosing regimen. Based on our findings that 9E9 remained bound at least up to 4 days *in vivo* both in spleen and tumor (Supplementary Figure 4), we administered the anti-FcγRIV antibody 1 day prior to anti-TIGIT:mIgG2a treatment and dosed the animals 3 times. As shown in Figure 2, the CT26 tumor-bearing mice treated with the 9E9 antibody followed by anti-TIGIT:mIgG2a displayed diminished anti-tumor efficacy when compared to the anti-TIGIT:mIgG2a + isotype control treatment group, demonstrating that the interaction of the anti-TIGIT:mIgG2a antibody with FcγRIV is of importance for optimal anti-tumor efficacy observed with that antibody (Figure 2).

### Tumors Are Established and Maintained in TIGIT Knockout Animals

**Figure 3:**
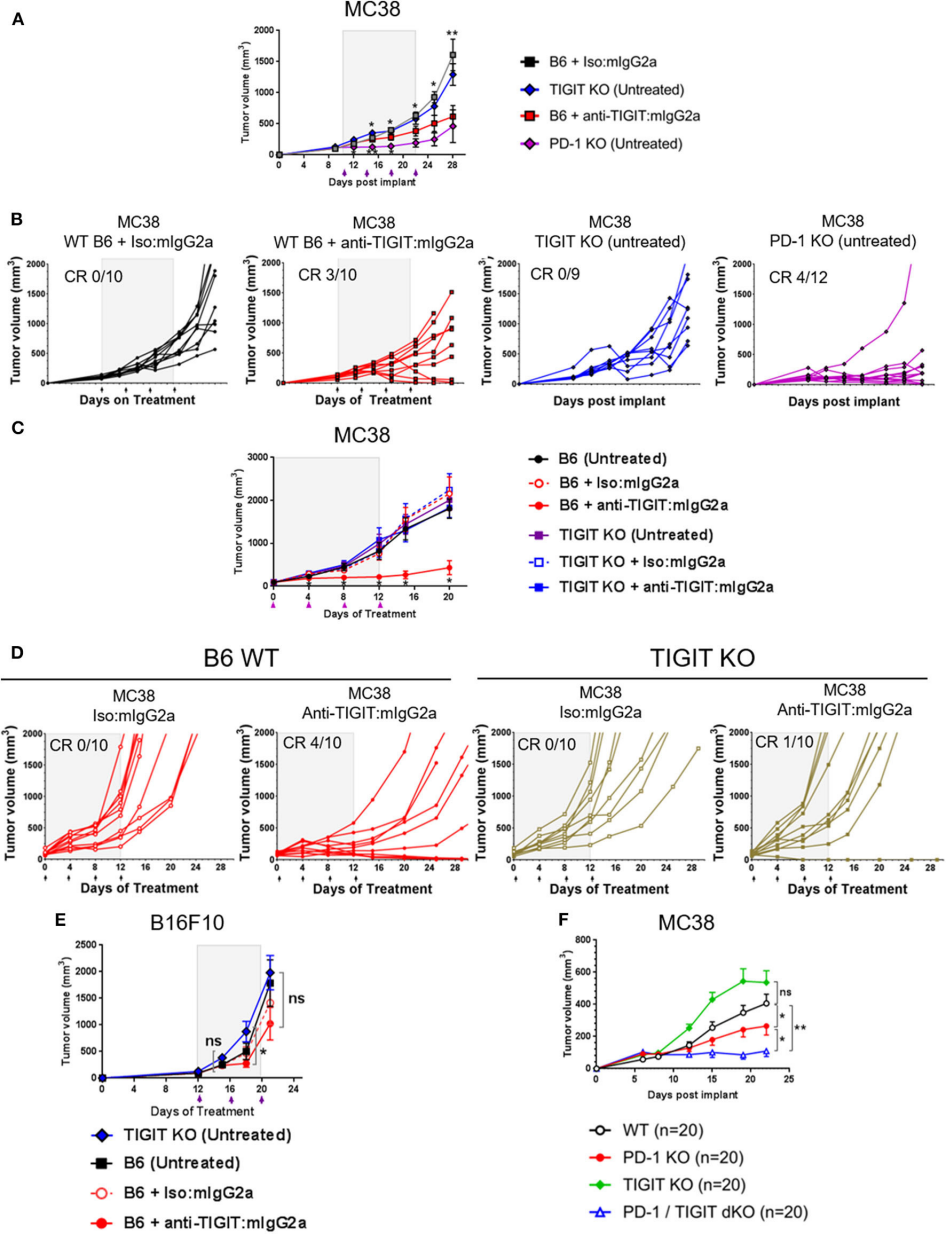
TIGIT-deficient mice do not spontaneously reject tumors. **(A,B,E)** Anti-tumor response by anti-TIGIT:mIgG2a antibody treatment is compared with spontaneous tumor rejections in TIGIT- or PD-1-deficient mice. Anti-TIGIT:mIgG2a antibody or isotype control antibody treatment was initiated when tumor [**(A,B)** with MC38; **(E)** with B16F10] was established on WT C57BL/6 mice at 101 mm^3^, whereas **(A,B,E)** TIGIT KO or **(A,B)** PD-1 KO mice did not get antibody treatments. Statistical analyses were done in a comparison with the anti-TIGIT:mIgG2a antibody treatment group. **(C,D)** Both antigen recognition and FcγR interaction *in vivo* are necessary for anti-tumor response by anti-TIGIT antibody. MC38 tumor was implanted and grown on either WT C57BL/6 or TIGIT KO mice. Mice with average 94 mm^3^ tumor were enrolled in the study for indicated antibody treatments (*n* = 10/group). **(F)** TIGIT/PD-1 dKO mice induce an enhanced spontaneous tumor rejection. Tumor volume was measured over time after MC38 tumor was implanted on WT C57BL/6, TIGIT KO, PD-1 PD, or TIGIT/PD-1 dKO mice (*n* = 20/group). ns, not significant; *p < 0.05; **p < 0.01. CR, complete responses.

Based on the observations that the anti-TIGIT: mIgG2a antibody is significantly more efficacious than an anti-TIGIT: mIgG1^*^ antibody and the demonstration that the interaction of the anti-TIGIT:mIgG2a with FcγRIV is required for full anti-tumor activity, we set out to investigate the ability of tumors to grow in TIGIT knockout (KO) mice. The TIGIT KO mice were generated using CRISPR/Cas9 technology in a pure C57BL/6 genetic background with a targeting strategy as described in the METHODS section and Supplementary Figure 5. We compared the tumor volumes of subcutaneous MC38 tumors on untreated TIGIT KO mice with those on WT mice treated with anti-TIGIT:mIgG2a antibody. We also included age, genetic background, and sex-matched PD-1-deficient mice (*Pdcd1*^−/−^ or PD-1 KO), in order to compare the tumor growth in TIGIT- vs. PD-1 KO mice. WT mice bearing MC38 tumors treated with an antagonist anti-TIGIT:mIgG2a antibody had 3/10 complete responses (Figures 3A–C), consistent with observations described earlier in the manuscript (Figure 1). This experiment has been repeated multiple times with subcutaneous MC38 tumors with the same outcome. Furthermore, treatment of TIGIT KO mice with the anti-TIGIT:mIgG2a antibody did not provide any further benefit to tumor growth inhibition, demonstrating the specificity of the anti-TIGIT antibody (Figures 3C,D). Because previous publications have reported a reduction of B16F10 tumor take in TIGIT KO mice (13), we also inoculated TIGIT KO mice with B16F10 cells subcutaneously but did not see anti-tumor response in our TIGIT KO mice (Figure 3E). Our data support and fortify the hypothesis that the Fc portion of the anti-TIGIT antibody is contributing to the anti-tumor efficacy of anti-TIGIT antagonist antibodies and that engagement of Fc receptor in addition to blocking the TIGIT:CD155 interaction is required for maximal anti-tumor efficacy. We also crossed the TIGIT KO mice to the PD-1 KO mice to generate PD-1/TIGIT double knockout (dKO) mice. Deficiency of PD-1 or/and TIGIT in respective mice has been confirmed by flow cytometry analysis (Supplementary Figure 6A). Interestingly, the PD-1/TIGIT dKO mice resulted in significantly less tumor take than the PD-1 KO mice. Given that the tumors were inoculated from the same cell culture at the same time and, and that in all instances the cell inoculations into WT mice yielded tumor growth, we are led to conclude that the underlying T-cell activation thresholds in the dKO mice differ significantly between WT and PD-1 KO situations, and that the dKO animals likely rejected the implanted tumors more efficiently (Figure 3F and (Supplementary Figure 6B).

### Anti-TIGIT:mIgG2a Antibodies Do Not Deplete Intratumoral Tregs

**Figure 4:**
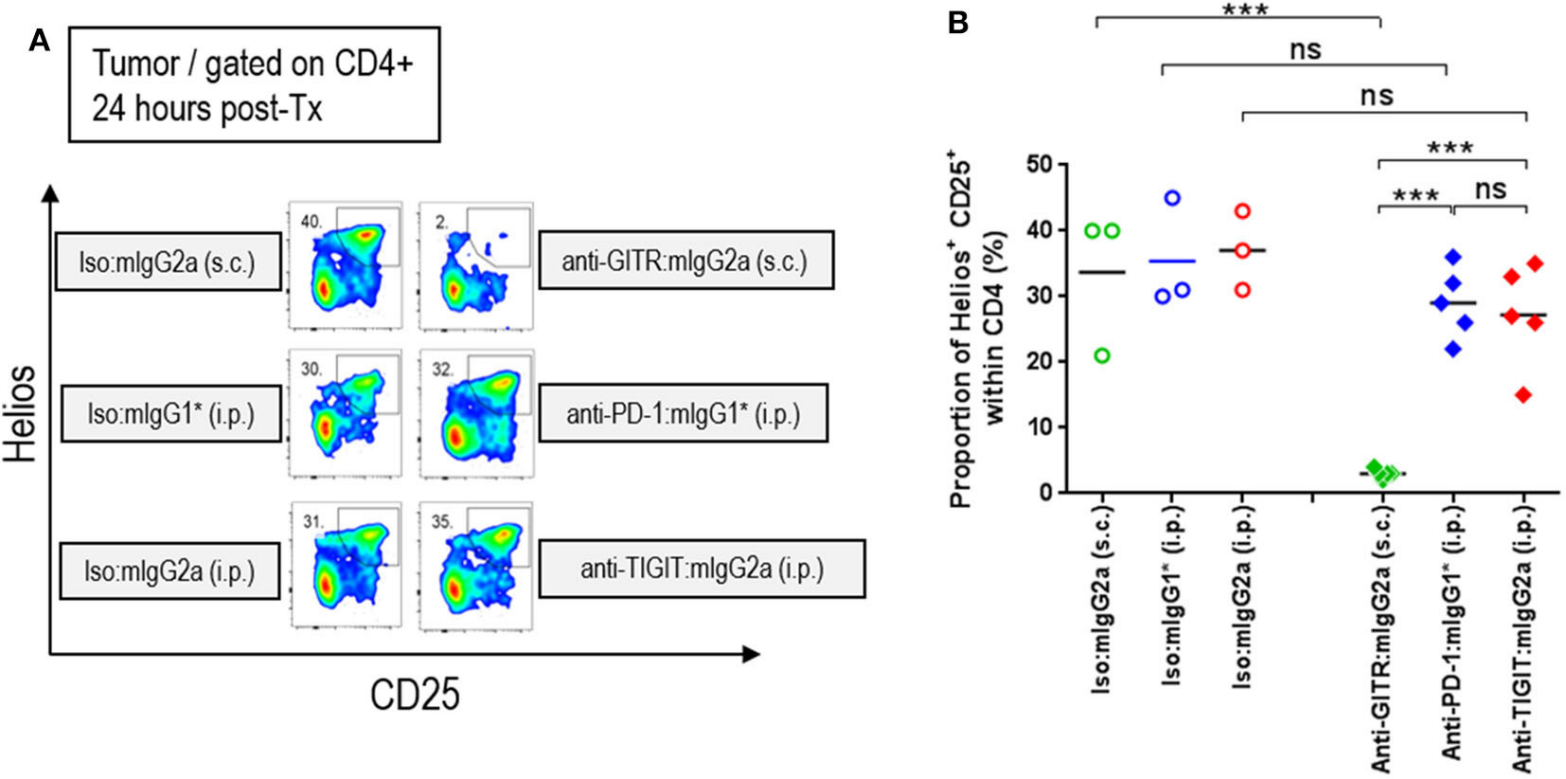
Efficacy of anti-TIGIT antibody is not mediated by intratumoral Treg depletion. In order to characterize the frequency of intratumoral Treg, CT26 tumor-bearing mice were injected with anti-GITR:mIgG2a (subcutaneous injection), anti-PD-1:mIgG1* (intraperitoneal injection), and anti-TIGIT:mIgG2a (intraperitoneal injection). Each of their isotype controls were also injected via the same routes as indicated. Twenty-four hours after each injection, the tumors were isolated and dissociated for flow cytometry analysis. **(A)** Representative plots and **(B)** frequencies of Helios^+^ CD25^+^ Tregs within intratumoral CD4 T cells from individual mice in each group are depicted. Data are representative of 2 independent experiments. ns, not significant; ***p < 0.005. i.p., intraperitoneal injection; s.c., subcutaneous injection.

It has been established in several mouse tumor models that an anti-CTLA-4 antibody on the mIgG2a isotype induces anti-tumor responses via depletion of intratumoral regulatory T cells via FcγRIV engagement (14). Similarly, an agnostic antibody to GITR was also shown to deplete intratumoral Tregs on the FcγR binding competent type mIgG2a isotype (15). Antibody-mediated cellular depletion occurs by antibody-induced cellular cytotoxicity (ADCC) or/and antibody-dependent cellular phagocytosis (ADCP). Because TIGIT is highly expressed on a subpopulation of Tregs that express Helios (16, 17), we hypothesized that an anti-TIGIT:mIgG2a may also deplete intratumoral Tregs that co-express TIGIT and Helios. To test this hypothesis, we dosed tumor-bearing animals with anti-TIGIT:mIgG2a antibodies as well as anti-GITR:mIgG2a (clone: DTA1) or anti-PD-1:mIgG1^*^ as positive or negative controls for intratumoral Treg depletion, respectively. Twenty four hours after injection, the frequency of CD25^+^ Helios^+^ population among CD4 T cells in the tumor was enumerated by flow cytometry. While the anti-GITR:mIgG2a group showed the expected drop in the CD4^+^ CD25^+^ Helios^+^ population in the tumor to below 5%, the anti-TIGIT:mIgG2a group did not show any signs of intratumoral Treg depletion, instead displaying around 25% of CD4^+^ T cells in the tumor being CD25^+^ Helios^+^ (Figure 4) which is similar to the anti-PD-1 group. These results indicate that, despite intratumoral Tregs expressing TIGIT at a relatively high density, the anti-TIGIT antibodies do not deplete intratumoral Tregs, leading us to conclude that their mechanism of anti-tumor efficacy does not involve to Treg depletion. Our observations are consistent with previously reported findings by both Johnston et al. (5) and Waight et al. (6). We next set out to investigate this alternative FcγR-dependent mechanism of tumor growth inhibition by anti-TIGIT antibody treatment.

### Treatment of Anti-TIGIT:mIgG2a Is Associated With Myeloid-Cell Activation

**Figure 5:**
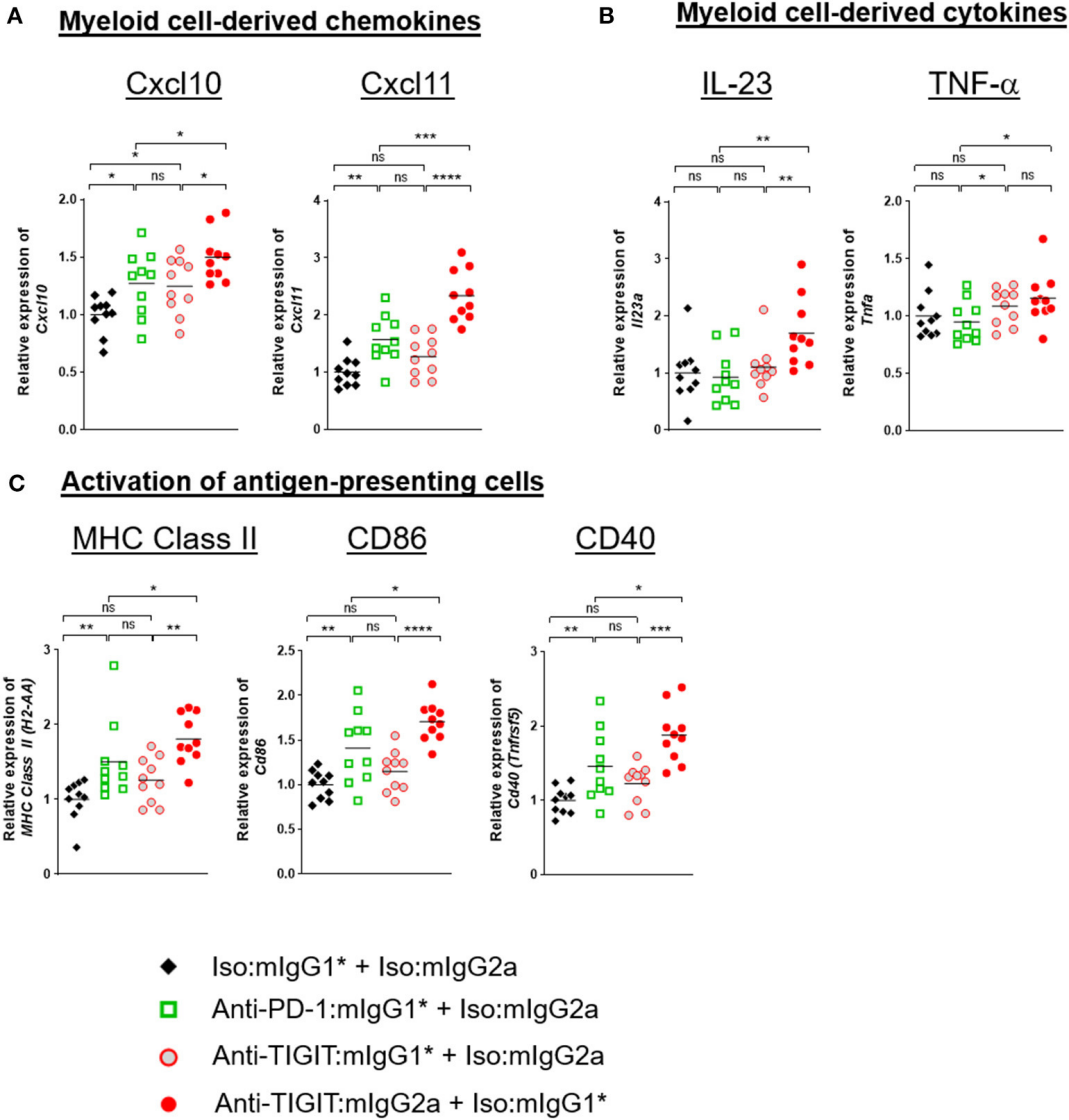
TIGIT blocking antibody requires FcγR-mediated myeloid-cell activation for anti-tumor response. CT26 tumor-bearing mice were injected with indicated antibody treatments two times in 4 days (day 0 and day 4). Four days after the second dose of each group (*n* = 10 per group), all the tumors were isolated and processed for real-time PCR. Relative gene expression profile of **(A)** Cxcl10 and Cxcl11, **(B)** IL-23, and TNF-α, **(C)** MHC class II, CD86, and CD40. The value of each gene expression is normalized with *Ubb*. Data are representative of 2 independent experiments. ns, not significant; *p < 0.05; **p < 0.01; ***p < 0.05; ****p < 0.001.

Because the level of TIGIT expression is higher in the TME than in the periphery (Supplementary Figure 2) and the TME contains many myeloid cells that express FcγRs, it is reasonable to hypothesize that anti-TIGIT:mIgG2a binds TIGIT first and then subsequently engages with surrounding FcγR-expressing myeloid cells through the Fc portion of the antibody. This IgG-Fc:FcγR binding may serve to activate myeloid cells, leading to an enhanced antigen presentation function (18) and chemokine and cytokine secretion (19). To better understand the mechanism of anti-TIGIT:mIgG2a efficacy, we designed an experiment to observe changes in whole tumors that were specific to treatment with an anti-TIGIT:mIgG2a antibody. Tumor-bearing mice were treated with anti-PD-1:mIgG1^*^, anti-TIGIT:mIgG1^*^, anti-TIGIT:mIgG2a, or isotype control antibodies through two treatment cycles 4 days apart. Four days after the second injection, we collected tumors and performed real-time PCR analysis on whole tumor samples. We found significant up-regulation of CXCL10, CXCL11 (Figure 5A), IL-23 and TNF-α (Figure 5B) when we treated with anti-TIGIT:mIgG2a as compared to anti-PD-1 or isotype controls. CXCL10 and CXCL11 are known to recruit T cells with the CXCR3 chemokine receptor, and IL-23 and TNF-α are indicative of inflammation by local myeloid cells (18). Additionally, we observed up-regulation of MHC class II, CD86, or CD40 the activation markers for antigen-presenting cells, specifically when TIGIT was engaged with an antibody that has an FcγR binding competent Fc (anti-TIGIT:mIgG2a) (Figure 5C). Treatment with an anti-TIGIT:mIgG1^*^ antibody caused increased gene expression only in some immune activation-related genes, including CXCL10 or TNF-α, suggesting that blocking the TIGIT:CD155 interaction may be leading to a lower level of immune activation *per se*.

### Enhanced Immune Activation in TME Through Combination of Anti-PD-1 and Anti-TIGIT Treatment Is Dependent on Presence of an FcγR-Binding Competent Isotype in the Anti-TIGIT Antibody

**Figure 6:**
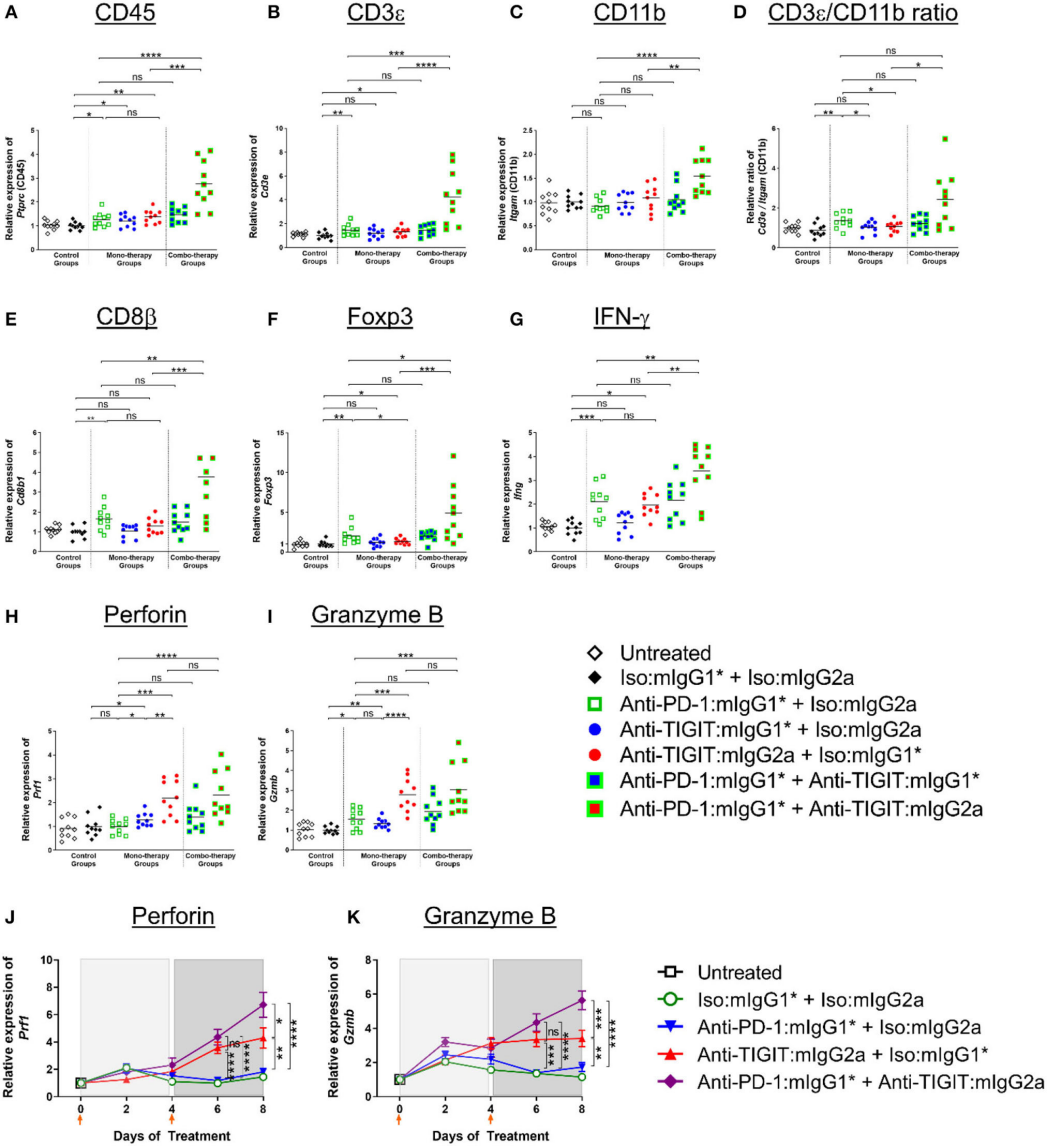
Enhanced immune activation in tumors by anti-PD-1 can be achieved only when anti-TIGIT antibody has a functional Fc. **(A–I)** In order to gain molecular insights of anti-PD-1 and anti-TIGIT combination for anti-tumor responses, anti-PD-1 in the presence or absence of anti-TIGIT with mIgG1* or mIgG2a isotype were therapeutically treated in CT26 tumor-bearing mice. Four days after the second dose of each group (n = 10 per group), all the tumors were isolated and processed for real-time PCR. Relative gene expression profile of **(A)** CD45, **(B)** CD3+, **(C)** CD11b, **(D)** CD3+/CD11b ratio, **(E)** CD8b, **(F)** Foxp3, **(G)** IFN-g, **(H)** Perforin, and **(I)** Granzyme B. **(J,K)** In an independent experiment, indicated antibody regimen were injected to CT26 tumor-bearing mice every 4 days. The whole tumors were harvested untreated (day 0), 2 days after first injection (day 2), 4 days after first injection (day 4), 2 days after second injection (day 6), and 4 days after second injection (day 8). Each symbol represents average and standard error of 10 tumors from each group at each time point for the analysis of **(J)** Perforin and **(K)** Granzyme B. Orange arrow heads indicate the time points of antibody treatments. ns, not significant; *p < 0.05; **p < 0.01; ***p < 0.005; ****p < 0.001.

Building on the above observation of myeloid cell activation in the TME (20), and in order to understand the significant combination benefit achieved in efficacy models by combining an anti-PD-1 antibody treatment with administration of an anti-TIGIT:mIgG2a antibody (Figures 1, 3), we set out to analyze gene expression of whole tumors 4 days after two doses of both single agents and combination antibody treatments in order to capture molecular characteristics of innate and early adaptive immune activities (for details, see legend of Figure 6). A significant increase of CD45 transcript was detected in the anti-PD-1 + anti-TIGIT:mIgG2a combination group when compared to single agent groups or the combination of anti-PD-1 with anti-TIGIT:mIgG1^*^ antibody, suggesting increased overall infiltration or/and proliferation of immune populations. We next compared the relative gene expression of T cells and myeloid cells in these tumors using their pan-cellular markers, CD3 (Figure 6B) and CD11b (Figure 6C), respectively. Both anti-PD-1 and anti-TIGIT:mIgG2a single agents induced a slight but significant increase of transcripts representative of total T cells (CD3ε) as compared to isotype control. However, a much larger increase was observed with the combination treatment with the anti-TIGIT:mIgG2a and anti-PD-1 antibodies. A similar observation was made for the CD11b transcript, suggesting myeloid infiltration, in the anti-PD-1 + anti-TIGIT:mIgG2a combination group when compared to single agents, combination of the anti-PD-1 + anti-TIGIT:mIgG1^*^, or isotype controls. When evaluating the ratio between CD3ε and CD11b transcripts, we noticed a proportionally much higher CD3ε transcript over CD11b cellular marker, suggesting a potential infiltration of T cells in the anti-PD-1 + anti-TIGIT:mIgG2a combination group, indicated by molecular markers (Figure 6D).

We next evaluated modulation of the CD8β and Foxp3 transcripts to gain insight into CD8 T cell and Treg responses. An increase of CD8 T cells (CD8β transcript) indicates infiltration or/and expansion of antigen-specific cytotoxic T cells, while Foxp3 is a marker for Tregs (21). Intratumoral Tregs also express both PD-1 and TIGIT (Supplementary Figure 2) and recognize tumor-specific antigens (22). A significant increase of the CD8β transcript occurred only when anti-TIGIT antibody had a functional Fc, suggesting a blocking of TIGIT:CD155 alone does not have an effect on CD8 T-cell expansion (Figure 6E). PD-1 blocking increased not only CD8 T cells but Tregs as well, while relative Foxp3 gene expression decreased when the tumor-bearing mice were treated with anti-TIGIT:mIgG2a, suggesting functional attenuation of intracellular Tregs or/and a proportional decrease of Treg population by other cell-types, such as myeloid cells as shown in Figure 6F and as suggested by others (7). It is important to note that the Foxp3 transcript was readily detectable in these tumors, suggesting that anti-TIGIT antibody, regardless of isotype, does not deplete Tregs in TME, corroborating our earlier observation (Figure 4). In summary, cell type-related transcripts, such as CD45, CD3ε, CD11b, CD8β, and Foxp3, were all most significantly up-regulated in the anti-PD-1 + anti-TIGIT:mIgG2a combination group (Figures 6A–F).

We next looked into the gene expression profile of key effector molecules such as IFN-γ, perforin, and granzyme B, which are known to play a critical role in mechanistic execution of an effective anti-tumor response (23). IFN-γ gene expression increased only in anti-PD-1, anti-TIGIT:mIgG2a monotherapy and anti-PD-1 + anti-TIGIT:mIgG2a combination therapy (Figure 6G) groups as compared to isotype controls and corroborates the anti-tumor efficacy we observe in multiple models with those treatments. In contrast and distinct from the IFN-γ modulation, perforin and granzyme B were up-regulated most strongly only by anti-TIGIT:mIgG2a or its combination with anti-PD-1 (Figures 6H,I).

Since our molecular analyses as presented in Figures 5, 6 represents a snapshot in time (*in vivo* study day 8) which is 4 days after the second antibody treatment, we extended our analysis to a time course evaluation of the dynamics of these mRNA expression profiles. Samples from CT26 tumors were collected on day 0 (untreated), day 2 (2 days after first injection), day 4 (4 days after first injection), day 6 (2 days after second injection), and day 8 (4 days after second injection) with each time point consisting of 10 distinct tumor samples per group to ensure a robust data set. The data at day 8 from this time course analysis showed remarkable consistency in the observed patterns to the data from our first experiment yielding the day 8 snapshot view as described and shown in Figures 6A–I, thus further validating and corroborating the previously acquired data. Figures 6J,K show representative datasets for perforin and granzyme B, respectively. The data demonstrates robust up-regulation of gene expression in the anti-PD-1 + anti-TIGIT:mIgG2a treatment group providing support to our hypothesis of successful activation of CD8 T cells as source of these lytic effector molecules. Interestingly, the FcγR binding competent anti-TIGIT antibody in a monotherapy treatment also demonstrated induction of mRNA for these markers, while its mIgG1^*^ counterpart was unable to effect such changes, consistent with our observations in tumor efficacy models (Figure 1). A full set of these time course expression profiles is provided in (Supplementary Figure 7) which also shows evaluation of chemokines (CXCL10, CXCL11) and transcripts indicative of activation of antigen-presenting cells (MHC class II, CD86, CD40). Again, strongest inductions are observed specifically when an FcγR-binding competent anti-TIGIT antibody (mIgG2a) is either used in monotherapy, or in combination with anti-PD-1. Overall, these data demonstrate a swift and lasting response of immune activation complete with up-regulation of effector molecules such as perforin and granzyme B which was most prominent and consistent in the anti-PD-1 + anti-TIGIT:mIgG2a combination treatment animals.

Our findings support a model that is suggestive of both T-cell activation as well as myeloid-cell signaling through FcγR engagement in an effective combination anti-cancer treatment of effector function enabled anti-TIGIT antibodies in combination with anti-PD-1. The mechanism is distinct from and complements the anti-PD-1 mechanism that is well-established as an effective clinical therapy in many indications.

## Discussion

Our observations reported in this work demonstrate that Fc engagement is necessary and required for anti-tumor efficacy of antagonist anti-TIGIT antibodies, and that blocking the TIGIT:CD155 interaction alone is not sufficient to confer anti-tumor efficacy of antagonist anti-TIGIT antibodies. While previous reports have demonstrated that Fc engaging antagonist anti-TIGIT antibodies are efficacious in preclinical tumor models, we herewith provide specific evidence that Fc engagement is required for efficacy based on data from tumor-bearing animals treated with non-Fc engaging anti-TIGIT antibodies and TIGIT KO mice, revealing some mechanistic insight into how Fc engagement of anti-TIGIT antibodies may be contributing to anti-tumor efficacy in the mouse.

As with the MC38 tumors, TIGIT KO mice did not demonstrate any reduction in tumor take when implanted with B16F10 cells as compared to WT mice (Figure 3D). This apparent discrepancy of our work with a published work (13) could be due to differences in specific experimental designs and in genetic (substrains of C57BL/6) or housing conditions. Another possible explanation is that both co-inhibitory receptors, PD-1 and TIGIT, perhaps participate in orchestrating thymic development in the WT animals and influence the elimination of T cells that react to autoantigens which potentially include “altered self” tumor neo-antigens. Further studies with TIGIT KO mice in multiple groups, more detailed analyses, and a possible head-to-head experiment to compare two TIGIT KO mice from each group will eventually clarify what underlies these differences.

As shown in Figures 5, 6, and (Supplementary Figure 7), our whole tumor gene profiling results (Figures 5, 6, Supplementary Figure 7) suggest that (i) an anti-TIGIT antibody on a mouse IgG2a isotype capable of engaging FcγRs appears to specifically induce markers indicative of myeloid cell activation, rather than depleting Tregs; (ii) blocking TIGIT:CD155 through an antibody incapable of effectively engaging FcγRs through the Fc portion of the antibody leads to only moderate inductions of a subset of these markers; and (iii) anti-TIGIT-mediated anti-tumor mechanism appears to be involving a pathway distinct from PD-1 blockade.

Based on parallels to the CTLA-4:CD28 costimulatory network and our own observation that anti-TIGIT efficacy is dependent on an FcγR engagement-competent antibody framework, we explored whether depletion of specific populations of cells (namely Tregs) that express TIGIT plays a role in anti-tumor efficacy. Contrary to previous work by others (14, 24), we do not observe a depletion of Treg as part of the anti-tumor mechanism of anti-TIGIT antibodies. Our data using the FcγR binding competent anti-TIGIT:mIgG2a are consistent with work by Waight et al. (6). who described anti-TIGIT antibodies with FcγR interaction driving anti-tumors response without a measurable depletion of intratumoral Tregs (Figures 1–4) (6). Here we significantly extend these observations and provide some mechanistic insight into how an anti-TIGIT antibody in the preclinical tumor models modulates APCs through FcγR engagement, resulting in chemokine release and activation of antigen-presenting cells (APCs) (Figures 5, 6). Because the level of TIGIT expression is higher in the TME than in the periphery (Supplementary Figure 2) and the TME contains many myeloid cells that express FcγRs, it is reasonable to hypothesize that anti-TIGIT:mIgG2a binds TIGIT first and then subsequently engages with surrounding FcγR-expressing myeloid cells through the Fc portion of the antibody. Such a sequence of events would likely take place because the binding of anti-TIGIT antibody to the TIGIT receptor is a high affinity interaction through the variable region of the antibody on an epitope, while the Fc portion of monomeric IgG binds FcγRs with a low affinity, creating a high avidity only when multiple IgGs are abundant in a proximity. This “avidity-based” binding may serve to activate myeloid cells, leading to an enhanced antigen presentation function (18) and chemokine and cytokine secretion (19). It has been well-established that stimulation via activating FcγRs is required for the production of cytokines such as IL-23, IL-27, IL-12, and TNF-α, and chemokines, like CXCL9, CXCL10, and MIP-1α, and furthermore the up-regulation of CD40 and CD86 on myeloid-derived APCs *in vitro* (18, 19).

CXCL9 and –10 are elements of what has been described as the CXCL9, –10, –11/CXCR3 axis that regulates immune cell migration, differentiation and activation, and thus have the potential to play a pivotal role in enhancing favorable clinical outcome of immune checkpoint inhibitor therapeutic interventions (23, 25). More specifically, CXCL9 has been reported to predominantly mediate lymphocyte infiltration to tumor sites and suppression of tumor growth (26). In concert with CXCL10 and –11, CXCL9 stimulates immune cells through Th1 polarization and activation leading to IFN-γ, TNF-α induction [both cytokines whose expression was also found to be up-regulated by anti-TIGIT:mIgG2a + anti-PD-1 combination treatment in our studies (Figures 5, 6)], and IL-2-mediated anti-tumor activity of cytotoxic T cells, NK cells and macrophages (27). Interestingly, and consistent with a role of these chemokines in tumor growth modulation, a critical role for their receptor CXCR3 has been identified by demonstration of enhanced tumor growth in CXCR3 KO mice with a syngeneic tumor model due to impaired CD8^+^ T cell migration (28). Similarly, Wendel et al. reported a significant reduction in tumor-infiltrating NK cells in CXCR3 KO animals, while CXCL10-controled NK cell recruitment correlated with tumor cell suppression and favorable prognosis (29). Furthermore, overexpression of CXCL9, –10 and –11 have all been reported to lead to various degrees of tumor growth inhibition in preclinical models, such as for example the oncolytic poxvirus-mediated delivery of CXCL11 in a mesothelioma disease model, causing induction of cytotoxic T lymphocytes in the TME and periphery (30). Maybe not surprisingly, the CXCL9, –10, –11 axis has also been linked to the PD-1 pathway, and preclinical models have shown a dependence of effective PD-1 treatment on functional CXCL9, –10, –11 signaling by demonstrating an inability of anti-PD-1 antibodies to shrink tumors in a CXCR3 KO background (28). Our observations of increased induction of CXCL10 and CXCL11 in the case of anti-TIGIT and anti-PD-1 combination treatment allows us to formulate a hypothesis where an anti-TIGIT antibody with FcγR binding-competent Fc moiety enhances anti-tumor activity through FcγR engagement of monocytes triggering the paracrine CXCL9, –10, –11/CXCR3 pathway leading to increased migration and activation of immune cells in the TME. It is important to point out that recent work by Chow and co-workers clearly support the significant of the CXCL9, –10, –11/CXCR3 axis for the success of anti-PD-1 therapy (31). Our work not only supports their findings but also demonstrates, for the first time, that this axis is even more amplified in anti-TIGIT therapy when the antibody carries a functional Fc than in anti-PD-1 therapy (Figure 5 and Supplementary Figure 7).

It is also worthy to note that an anti-TIGIT:mIgG2a antibody induces a more robust up-regulation of perforin and granzyme B gene expression as compared to an anti-PD-1 antibody in our studies - a feature that could differentiate it from treatment with anti-PD-1 antibodies (Figures 6H–K) - suggesting that an anti-TIGIT antibody boosts cytotoxic pathways of TILs distinctive and separate from the PD-1 blockade. This is a significant finding because it is well-established that some tumors evolve to escape immune pressure by avoiding the IFN-γ-mediated immune surveillance (32). One possibility is that anti-TIGIT blockade provides complementary anti-tumor activities to the IFN-γ pathway by reinforcing the granzyme B and perforin activations (Figure 6 and Supplementary Figure 7). The underlying nature of this mechanistic difference between TIGIT- and PD-1-targeting approaches remains to be further elucidated.

Additionally, our studies reveal a significant activation of APCs in the TME upon FcγR engagement by an administered anti-TIGIT antibody. This sets the stage for possible enhanced antigen presentation that can be achieved when tumor antigens are released and become available in the TME. It will be interesting to see whether any combination of anti-TIGIT (with a functional Fc) with an agent inducing immunogenic cell-death could further potentiate the efficacy of anti-tumor response *in vivo*.

In summary, we have demonstrated that antagonistic anti-TIGIT antibodies with an FcγR-engaging isotype induce strong anti-tumor efficacy both when administered as a monotherapy and in combination with a PD-1 blockade in preclinical tumor models. The observed anti-tumor activity is far reduced when using the same anti-TIGIT antibodies but with an isotype devoid FcγR engagement. Consistent with these observations we have also demonstrated that blocking FcγR engagement results in significantly reduced anti-tumor efficacy of anti-TIGIT antibodies with functional Fc. We furthermore have shown that FcγR engagement of anti-TIGIT antibodies drives persistent activation of APCs, and that blocking the TIGIT:CD155 interaction leads to elevated expression of granzyme B and perforin, which mechanistically sets it apart from anti-PD-1 treatments in preclinical mouse tumor models. Consistent with our data, recent work by Waight et al. (6) demonstrated *in vitro* that a maximal amount of IL-2 was produced when human PBMCs are stimulated in the presence of anti-human TIGIT antibody of the human IgG1 isotype (mIgG2a equivalent), and that availability of human FcγRIIIA (mouse FcγRIV equivalent) is required for the most robust T-cell activation in this *in vitro* setting (6).

While generally accepted that caution has to be applied when attempting to translate this biology from preclinical species to the human situation, it will be very interesting and revealing to follow the outcome of clinical trials currently underway with both, FcγR-binding competent (IgG1) and binding-reduced (IgG4 of IgG1 mutant) isotypes of anti-human antagonist TIGIT antibodies which may provide insight into whether anti-human TIGIT antibodies also require FcγR-binding competent isotypes for optimal anti-tumor responses in the clinic where several anti-human TIGIT antibodies are currently evaluated in patients with solid tumors as a monotherapy or in combination with anti-PD-(L)1.

## Methods

### Mice

Wild-type female mice (6–8 weeks old) were purchased from The Jackson Laboratory (C57BL/6J strain) and Taconic (BALB/cAnTac and C57BL/6NTac strains). PD-1-deficient mice were generated as described previously (9). The TIGIT-deficient mice were generated at Taconic Biosciences Inc for Merck Research Laboratories. The targeting strategy allows the generation of a conditional and a constitutive Knock-out of the *Tigit* gene (NCBI gene ID: 100043314). The TIGIT knockout mouse was generated by flanking exons 2 and 3, encoding the extracellular domain (including the Ig-like V-type domain), with LoxP sequences. Positive selection markers for LoxP insertion were flanked by neomycin and puromycin resistance gene cassettes and were inserted into intron 1 and intron 3, respectively. The targeting vector was generated using genomic DNA from BAC clones and was transfected into a C57BL/6NTac ES cell-line. Homologous recombinant ES cell clones were isolated using double positive (Neo^R^ and Puro^R^) selection. Conditional KO allele was obtained after *in vivo* Flp-mediated removal of the selection markers and the constitutive KO mice were generated by crossing to Rosa26-Cre deleter mice. Homozygous and WT littermate control mice were used in the experiments.

### Antibodies for *in vivo* Experiments

Anti-murine PD-1 (clone DX400) and mouse isotype control antibodies are described previously (Hossain). Anti-murine TIGIT monoclonal antibodies were generated by immunizing xxxx rats with recombinant mouse TIGIT protein in Merck & Co., Inc. Screened rat-derived monoclonal antibody clones were screened, and selected clones, including clones 18G10 and 11A11, were subsequently murinized with mouse IgG isotypes, as indicated in the main text. Low endotoxin, azide-free anti-mouse FcγRIV antibody (clone 9E9) and its isotype control (Armenian hamster IgG, clone HTK888) were purchased from Biolegend.

### Cell-Lines

Mouse syngeneic tumor cell-lines, MC38, CT26, and B16F10, were originally purchased from ATCC and maintained and expanded based on its guideline at core laboratories of Merck & Co., Inc. A quality control (QC) test that includes the cell authentication and Mycoplasma testing was done using a Polymerase Chain Reaction method for each expanded batch of each cell-line (several times each year). Data from all *in vivo* experiments were generated by cell-lines that had passed QC tests.

### *In vivo* Experiments

Mice were injected subcutaneously with MC38 (1 × 10^6^) or CT26 (0.3 × 10^6^) cells into the lower right flank. *In vivo* antibody treatments were carried out as indicated. Growth of implanted tumor was recorded by tumor volume as calculated by a formula: 0.5 × length × width^2^, where the length was the longer dimension. Tumor growth inhibition (TGI) was calculated using the formula: [(C_t_ – C_0_) – (T_t_ – T_0_)]/(C_t_ – C_0_) × 100, where C_t_ = the mean tumor volume of the control group at time (t); C_0_ = the mean tumor volume of the control group at t_0_; T_t_ = mean tumor volume of the treatment group at t; and T_0_ = mean tumor volume of the treatment group at t_0_.

### Flow Cytometry

Tumor single-cell suspension was prepared by homogenizing dissected tumors using GentleMACS dissociator (Miltenyi Biotec) in ACK buffer (Lonza Bioscience) to lyse red blood cells immediately. Centrifugated cell pellets were resuspended in RPMI-1640 (Sigma) with 5% fetal bovine serum (FBS) and subsequently filtered through 70 μm cell strainers (Fisher Scientific). Prepared single-cell suspension was stained with antibodies against murine CD45 (clone: 30-F11; BD Biosciences), CD4 (clone: RM4-5; Biolegend), CD8β (clone: H35-17.2; eBioscience), CD25 (clone: PC61, BD Biosciences), PD-1 (clone: RMP1-30; eBioscience), TIGIT (clone: 1G9, Biolegend), and Helios (clone: 22F6; Biolegend) in a FACS buffer (2% FBS, 1 mM EDTA, and 0.1% NaN_3_ in DPBS) with empirically determined optimal antibody concentrations. The Data from sample was acquired by BD LSRFortessa (BD Biosciences) and the acquired data were analyzed with Flowjo software (BD Bioscience).

### Whole Tumor Gene Expression Profiling by TaqMan Assay

For analysis of gene expression, whole tumors were isolated from the animals and snap-frozen in liquid nitrogen, tissues were homogenized and lysed, and RNA was isolated. DNase-treated total RNA was reverse transcribed using QuantiTect Reverse Transcription (QIAGEN) according to the manufacturer's instructions. The gene-specific primers were obtained commercially from Thermo Fisher Scientific. Gene-specific pre-amplification was done on 10 ng cDNA according to the manufacturer's instructions (Fluidigm). Real-time qPCR was then performed on the Fluidigm Biomark using 2 unlabeled primers at 900 nM each, along with 250 nM FAM-labeled probe (Thermo Fisher Scientific) and TaqMan Universal PCR Master Mix with uracil-N-glycosylase (UNG). Samples and primers were run on a Fluidigm 96 instrument. The RNA level of Ubiquitin B (encoded by *Ubb*) were measured to use for normalization of the data analysis by the ΔCt method. Using the mean cycle threshold value for *Ubb* and the gene of interest for each sample, the equation 1.8 (Ct *Ubb* – Ct gene of interest) × 10^4^ was used to obtain the normalized values. Data were depicted as indicated in each figure. The average FC of treated over untreated samples was calculated, and Students' *t*-test analysis was performed to determine *P*-values.

### Statistical Analyses

One or two-way unpaired Students' *t*-test was used to assess the statistical significance of groups in comparisons using GraphPad Prism 7 (GraphPad Software).

### Approval of Animal Studies and IACUC Guideline

All animal procedures were approved by the IACUC of Merck & Co., Inc., (Kenilworth, NJ, USA) in accordance with Association for Assessment and Accreditation of Laboratory Animal Care (AAALAC) guidelines. It is noteworthy to indicate that we practice a very strict guidelines on handling tumor-bearing mice, which tries to avoid inhumane suffering and death of the animals due to excessive tumor burden. We implement an internal guideline to euthanize the animals when the measured tumor volume exceeds 2,000 mm^3^.

## Data Availability Statement

The authors acknowledge that the data presented in this study must be deposited and made publicly available in an acceptable repository, prior to publication. Frontiers cannot accept a article that does not adhere to our open data policies.

## Ethics Statement

The animal study was reviewed and approved by IACUC of Merck & Co., Inc, (Kenilworth NJ, USA).

## Author Contributions

J-HH, SP, TR, EP, DL, WS, and SW conceived the ideas. J-HH, MC, JG, HW, MB, RU, TR, and DL performed experiments. J-HH, WS, and SW wrote the manuscript. All authors participated in the analysis and discussion of the data.

## Conflict of Interest

All authors are employees and shareholders of Merck & Co.
