# Corrigendum: Effective Anti-tumor Response by TIGIT Blockade Associated With Fc*γ*R Engagement and Myeloid Cell Activation

**DOI:** 10.3389/fimmu.2020.615755

**Published:** 2020-11-18

**Authors:** Jin-Hwan Han, Mingmei Cai, Jeffery Grein, Samanthi Perera, Hongmei Wang, Mike Bigler, Roenna Ueda, Thomas W. Rosahl, Elaine Pinheiro, Drake LaFace, Wolfgang Seghezzi, Sybil M. Genther Williams

**Affiliations:** Merck & Co. Inc., Kenilworth, NJ, United States

**Keywords:** TIGIT, FcγR, myeloid cells, combination cancer immunotherapy, costimulatory molecules, immune checkpoint blockade

In the original article, there was a mistake in **Figure 6** as published. While upgrading the figure with a high resolution for publication, the “on-figure” legend describing each experimental group was depicted inconsistently from the rest of the article. The corrected **Figure 6** appears below.

The authors apologize for this error and state that this does not change the scientific conclusions of the article in any way. The original article has been updated.

**Figure 6:**
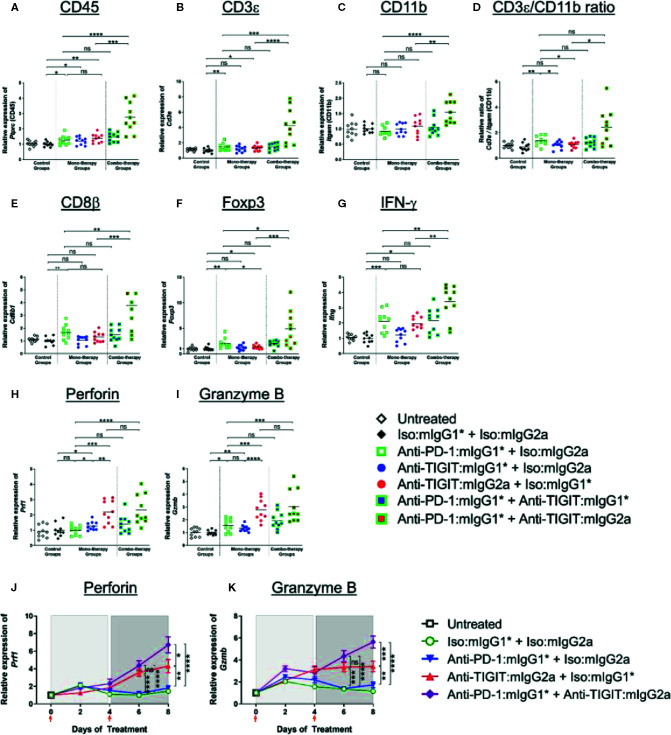
Enhanced immune activation in tumors by anti-PD-1 can be achieved only when anti-TIGIT antibody has a functional Fc. **(A–I)** In order to gain molecular insights of anti-PD-1 and anti-TIGIT combination for anti-tumor responses, anti-PD-1 in the presence or absence of anti-TIGIT with mIgG1* or mIgG2a isotype were therapeutically treated in CT26 tumor-bearing mice. Four days after the second dose of each group (n = 10 per group), all the tumors were isolated and processed for real-time PCR. Relative gene expression profile of **(A)** CD45, **(B)** CD3+, **(C)** CD11b, **(D)** CD3+/CD11b ratio, **(E)** CD8b, **(F)** Foxp3, **(G)** IFN-g, **(H)** Perforin, and **(I)** Granzyme B. **(J, K)** In an independent experiment, indicated antibody regimen were injected to CT26 tumor-bearing mice every 4 days. The whole tumors were harvested untreated (day 0), 2 days after first injection (day 2), 4 days after first injection (day 4), 2 days after second injection (day 6), and 4 days after second injection (day 8). Each symbol represents average and standard error of 10 tumors from each group at each time point for the analysis of **(J)** Perforin and **(K)** Granzyme B. Orange arrow heads indicate the time points of antibody treatments. ns, not significant; *p < 0.05; **p < 0.01; ***p < 0.005; ****p < 0.001.

