# Supplementary material for: Effective Anti-tumor Response by TIGIT Blockade Associated With FcγR Engagement and Myeloid Cell Activation
Source: Front Immunol. 2020 Oct 7;11:573405. doi: 10.3389/fimmu.2020.573405 (PMC7577118; doi:10.3389/fimmu.2020.573405)
Supplement: Supplementary file 1 [file Data_Sheet_1.PDF]

Supplementary Material

Effective anti-tumor response by TIGIT blockade
associated with FcγR engagement and myeloid cell activation

Supplementary Figure 1

A

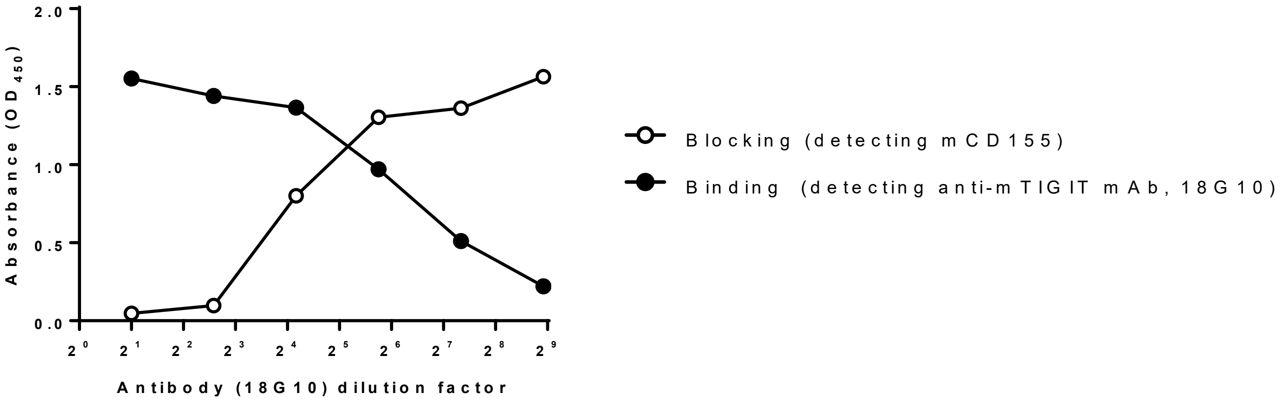

B

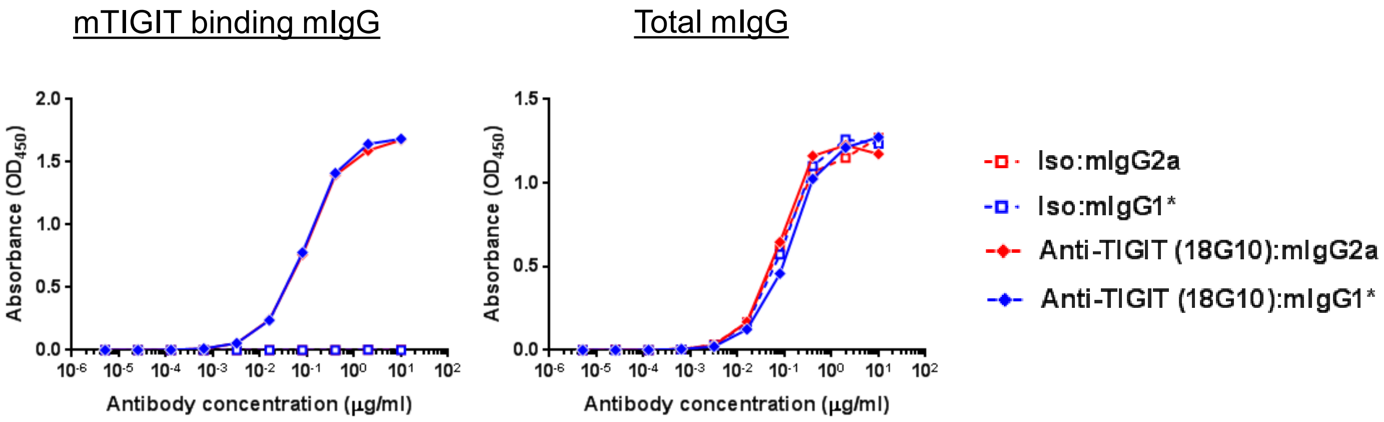

Supplementary Figure 1. Identification and characterization of anti-mouse TIGIT monoclonal antibody 18G10.

(A) Binding of anti-mouse TIGIT, clone 18G10, was measured by a cell-based ELISA using mouse TIGIT-overexpressing CHO cells (mTIGIT/CHO). 18G10 was serially diluted as indicated. After 30 min. of incubation, unbound 18G10 antibody was washed out, and the bound 18G10 antibodies were detected using an anti-mouse IgG reagent (closed circles). When the 18G10 was bound in the indicated serial dilutions on mTIGIT/CHO, 8 μg/ml recombinant mouse CD155:hIgG1-Fc (rmCD155) was added to the plate in order to verify whether 18G10 blocked the interaction of rmCD155 with mTIGIT/CHO. After 40 min. of incubation, unbound recombinant CD155 was washed out, and the bound recombinant CD155 was detected by anti-human IgG reagent (open circles).

(B) Fc variants of an anti-mouse TIGIT monoclonal antibody clone 18G10 did not change their specific binding to recombinant mouse TIGIT protein. Chimeric anti-TIGIT (18G10):mIgG2a, anti-TIGIT (18G10):mIgG1-[D265A], and isotype controls with mIgG2a or mIgG1-[D265A] isotype were loaded to ELISA plates coated with 1 μg/ml of (left panel) recombinant mouse TIGIT protein or (right panel) goat anti-mouse IgG-Fc antibody. Bound antibodies to each plate were detected. Note: mIgG1* indicates mIgG1-[D265A]

Supplementary Figure 2

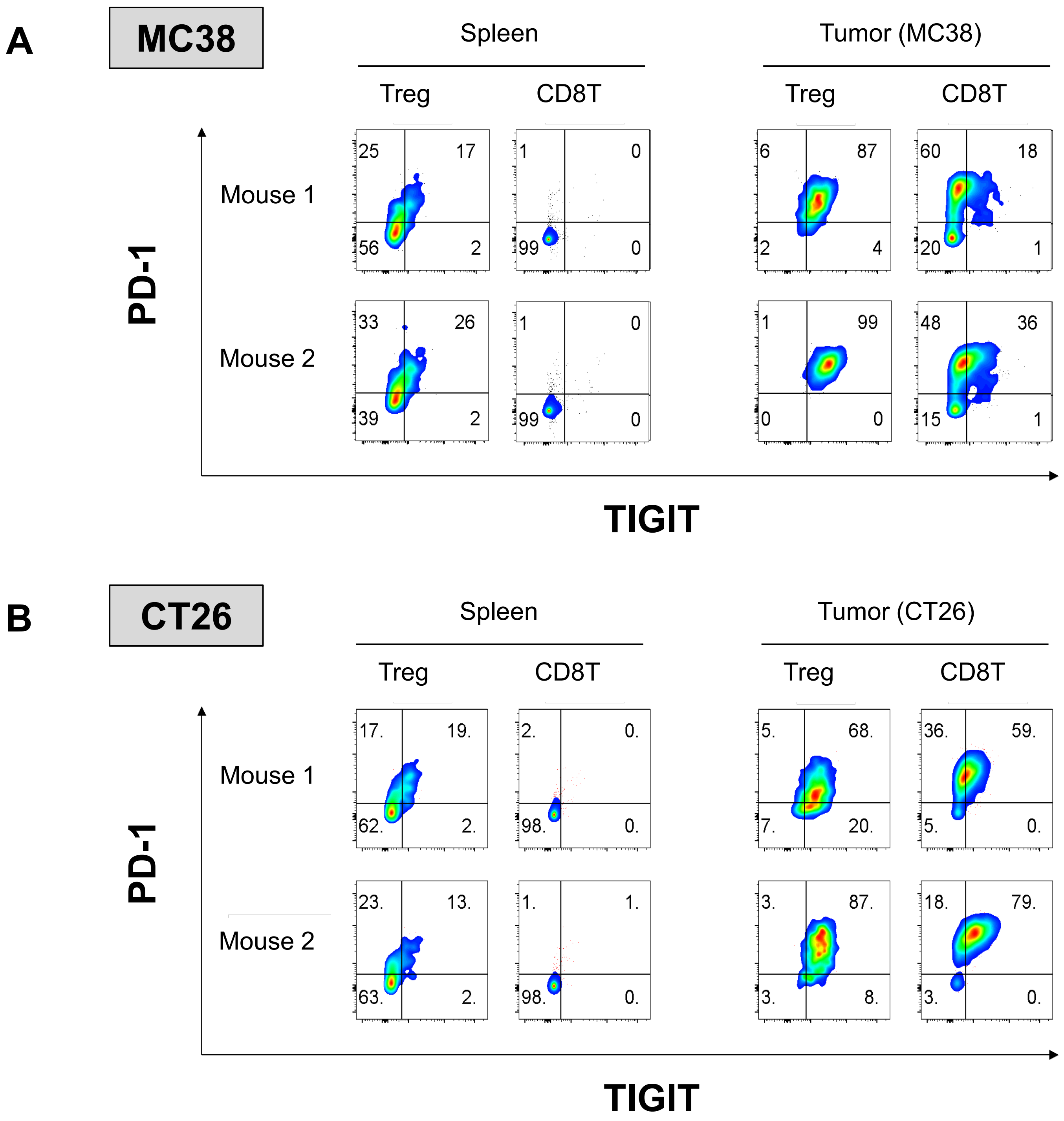

Supplementary Figure 2. Surface expression of PD-1 and TIGIT on CD8 and Tregs in spleen and MC38 or CT26 tumor.

Spleens and tumors from (A) MC38 or (B) CT26 tumor-bearing mice were isolated and dissociated for flow cytometry to stain for PD-1 and TIGIT on Treg and CD8 T cells. The PD-1 and TIGIT expression profile on Tregs ($CD4^+$ $Foxp3^+$) or $CD8\alpha^+$ within $CD45^+$ $CD11b^-$ $TCR\beta^+$ $CD3^+$ population is depicted. **, P< 0.01 (Mann-Whitney test).

Supplementary Figure 3

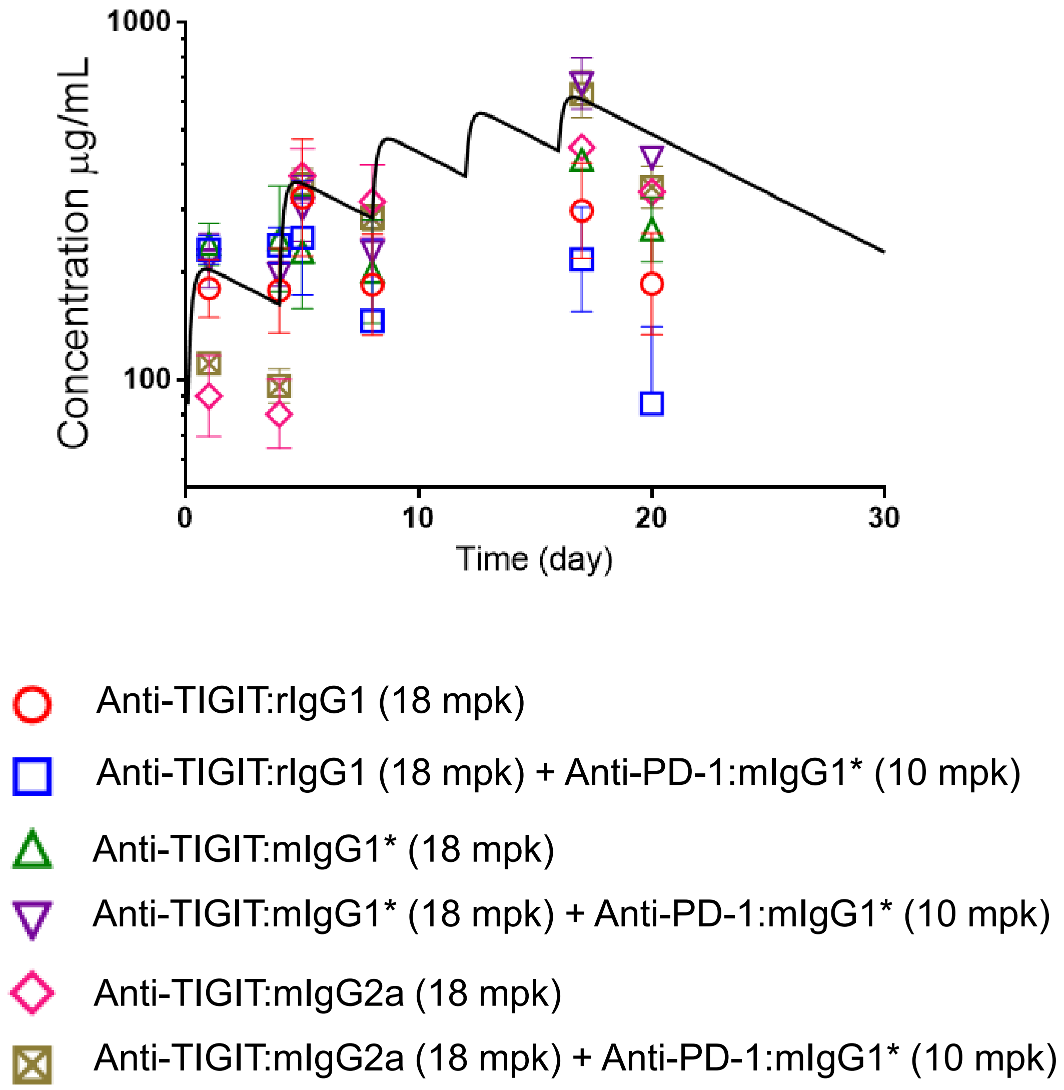

Supplementary Figure 3. In vivo drug exposure of anti-TIGIT antibody with various isotypes.

CT26 tumor-bearing mice were injected with 18 mpk intraperitoneally anti-TIGIT antibody (clone 18G10) with rat IgG1 (rIgG1), mouse IgG1-[D265A] (mIgG1*), and mouse IgG2a (mIgG2a) as monotherapies or in combinations with 10 mpk anti-PD-1:mIgG1* as indicated above. Serum samples were collected at day 1 (1 day after the first dose), day 4 (4 days after the first dose), day 8 (4 days after the second dose), day 16 (4 days after the fourth dose), and day 20 (8 days after the fourth dose, terminal). The serum samples were serially diluted and loaded on a bioassay plate coated with recombinant mouse TIGIT proteins to quantitate the amounts of circulating anti-TIGIT antibodies with various isotypes.

Supplementary Figure 4

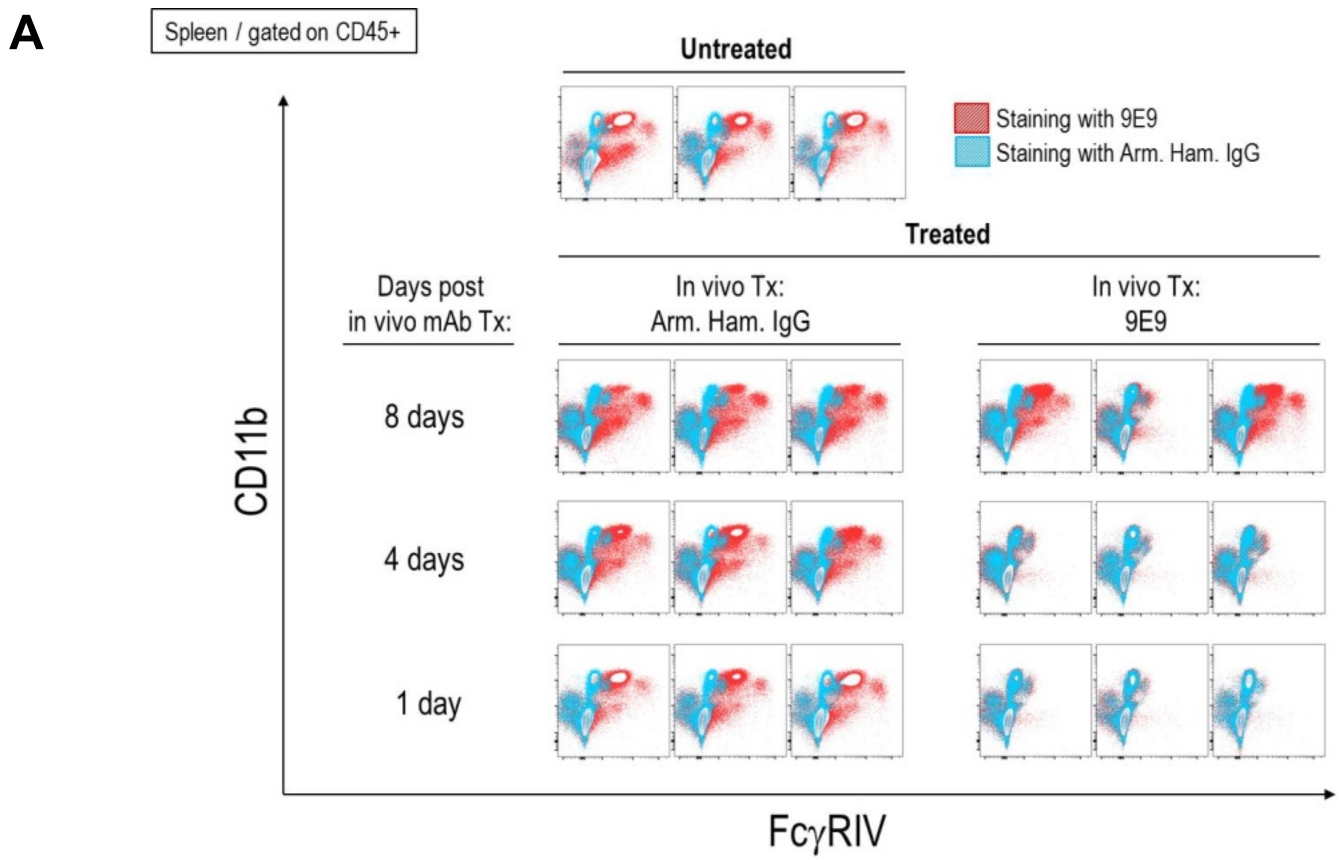

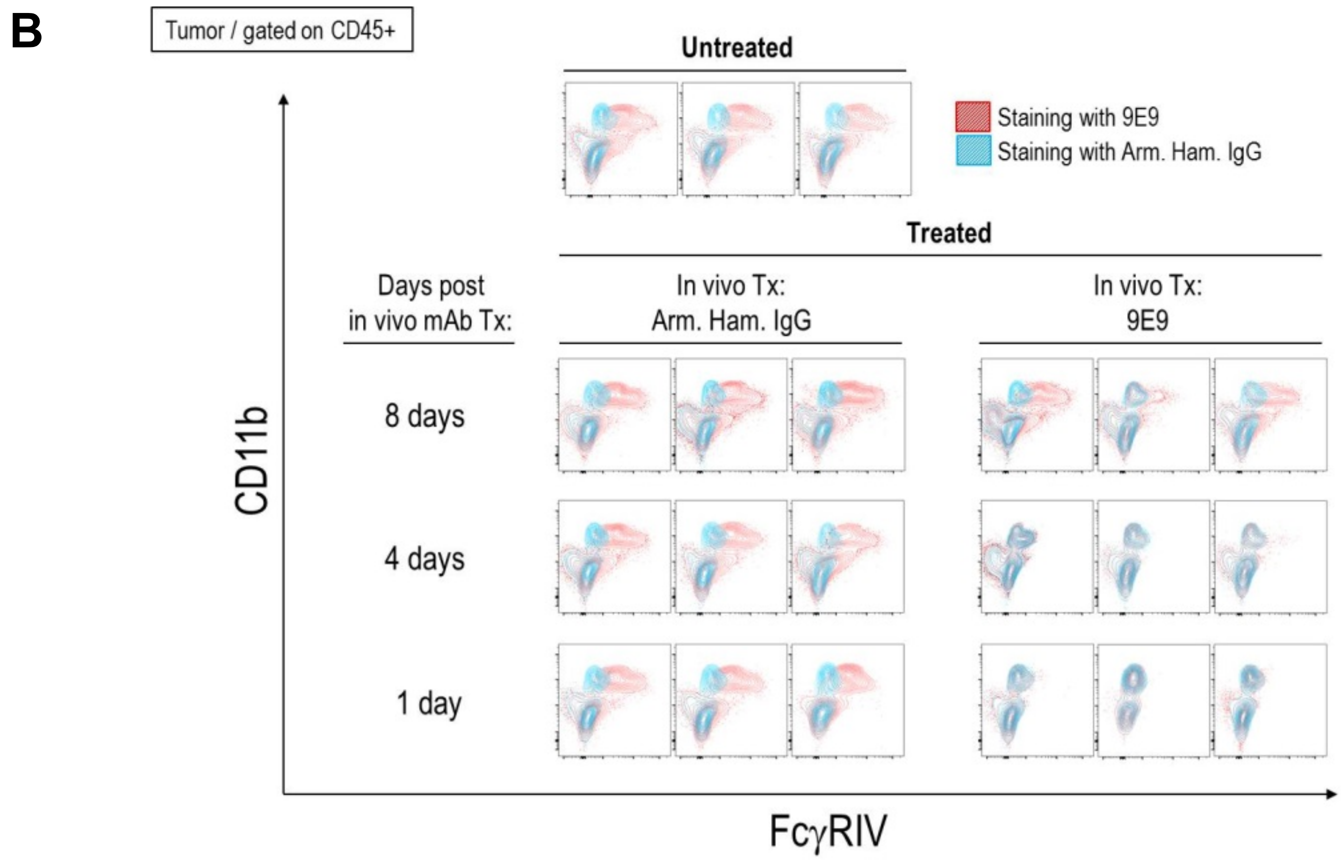

Supplementary Figure 4. Duration of 9E9 binding *in vivo*.

MC38 tumor-bearing mice were injected with 10 mpk of isotype control (Armenian Hamster IgG) or anti-mouse FcγRIV antibody (9E9) intraperitoneally for 1 day, 4 days or 8 days. Both (A) spleen and (B) tumor were isolated and dissociated to make single-cell suspensions to stain for flow cytometry. Fluorochrome-conjugated 9E9 was included in the staining cocktail to stain FcγRIV. Plots are shown after gating the CD45+ cells. As expected, FcγRIV is expressed in $CD11b^+$ myeloid cells in (A) spleen and (B) tumor. Up to day 4, the FcγRIV was not stained due to epitope blocking by previously injected 9E9 to the animals. The functional blocking effect of 9E9 was published previously in (Kaneko et al., (2006) 203 (3):789-797).

Supplementary Figure 5

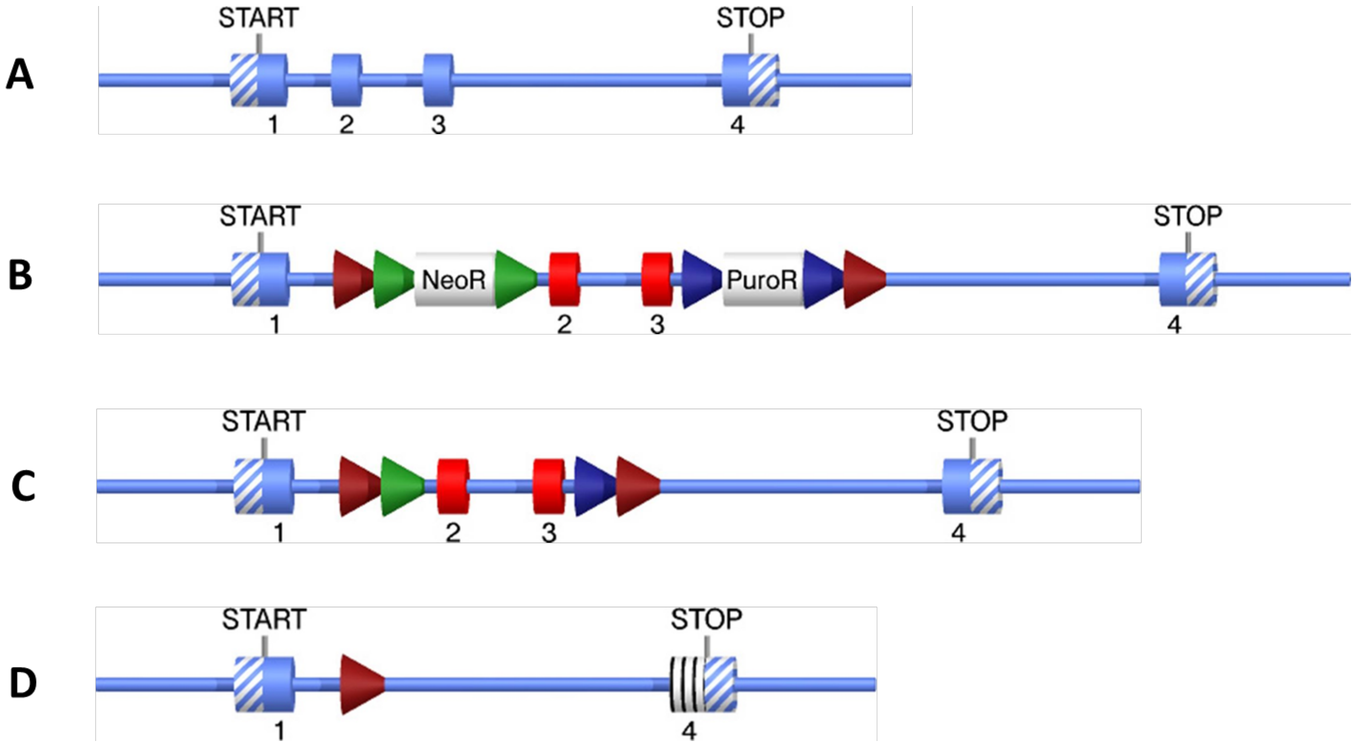

Supplementary Figure 5. Targeting strategy of the TIGIT KO mice.

(A) Mouse genomic locus of the *Tigit* gene. (B) Targeted allele after homologous recombination in ES cells. (C) Conditional KO allele after Flp recombinase mediated removal of the positive selection markers. (D) Constitutive *Tigit* KO allele after cre mediated recombination resulting in deletion of exons 2 and 3 and a frameshift in exon 4. *Tigit* coding exons 1-4 and translational start and stop codons are depicted. Red triangle: LoxP site; green triangle: FRT site; blue triangle: F3 site; Neo^R: Neomycin resistance gene cassette; $Puro^R$: Puromycin resistance gene cassette.

Supplementary Figure 6

A

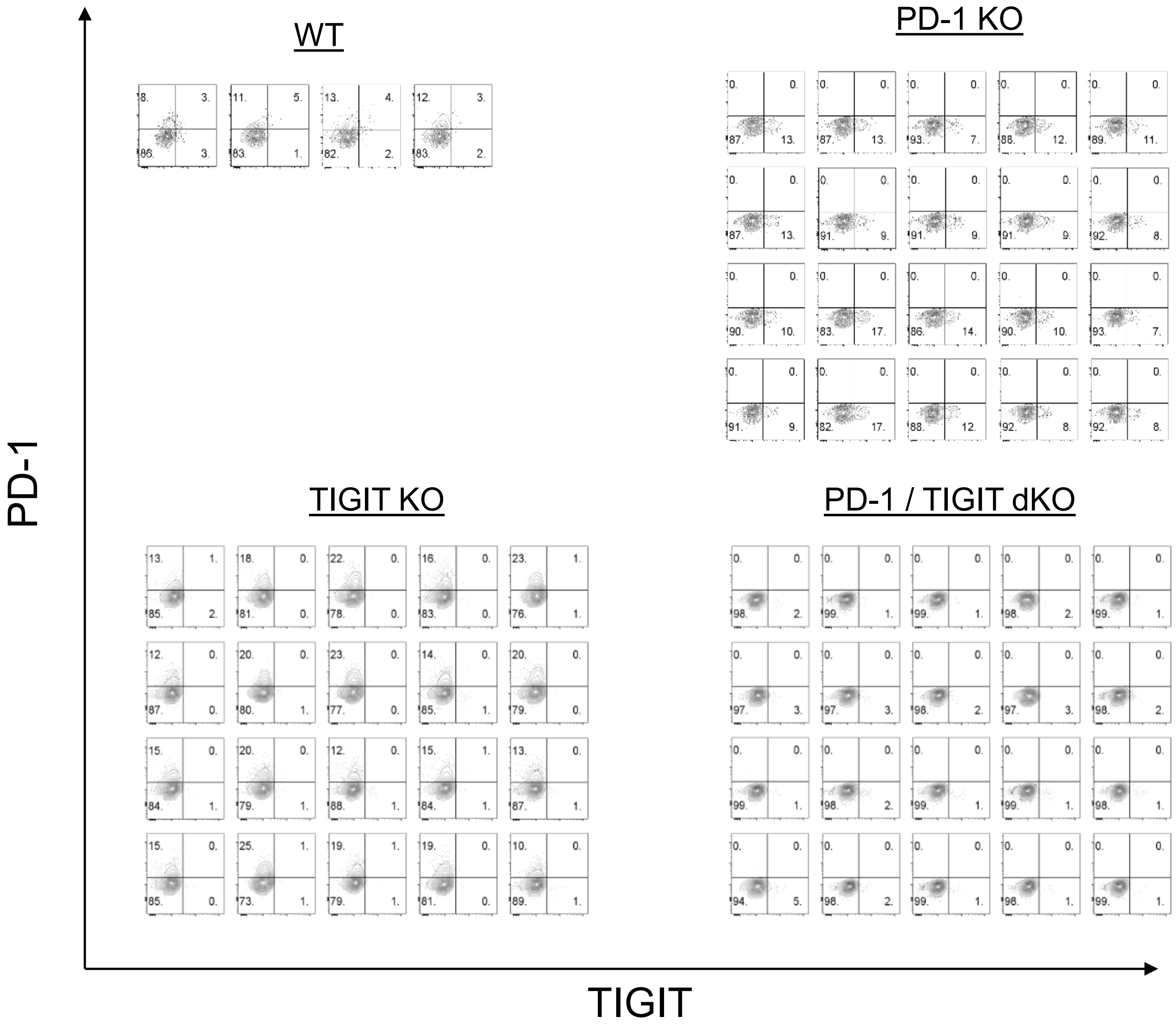

Supplementary Figure 6

B

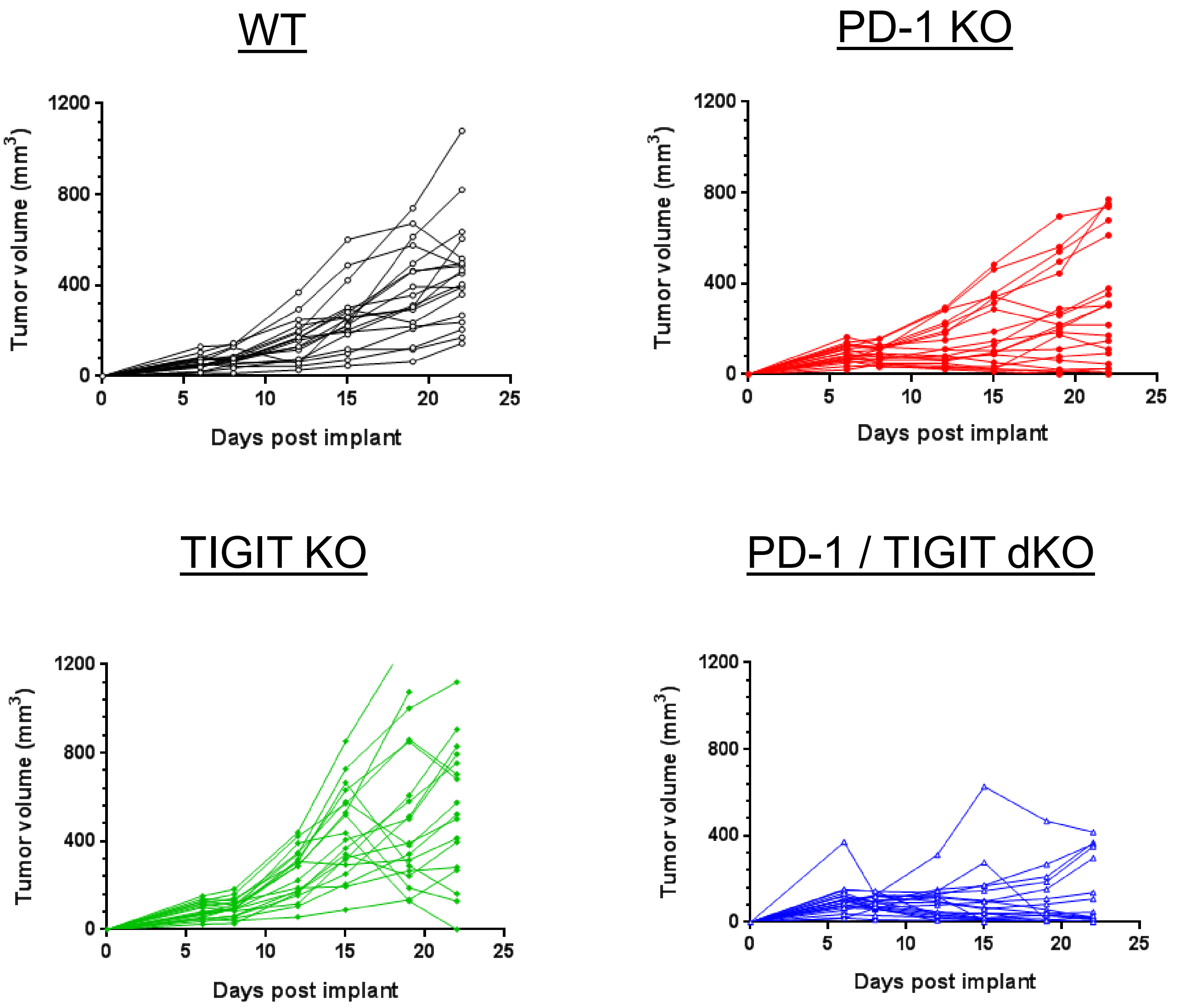

Supplementary Figure 6. TIGIT-deficient mice do not spontaneously reject tumors.

(A) Expression of surface PD-1 and TIGIT expression in blood cells was determined by flow cytometry. The expression of PD-1 and TIGIT is depicted gated on CD4^{+} cells in the blood. MC38 syngeneic tumor cells ($1x10^{6}$) were implanted to 20 mice of WT, PD-1 KO, TIGIT KO, and PD-1/TIGIT dKO, respectively. Tumor volume was measured twice a week.

Supplementary Figure 7

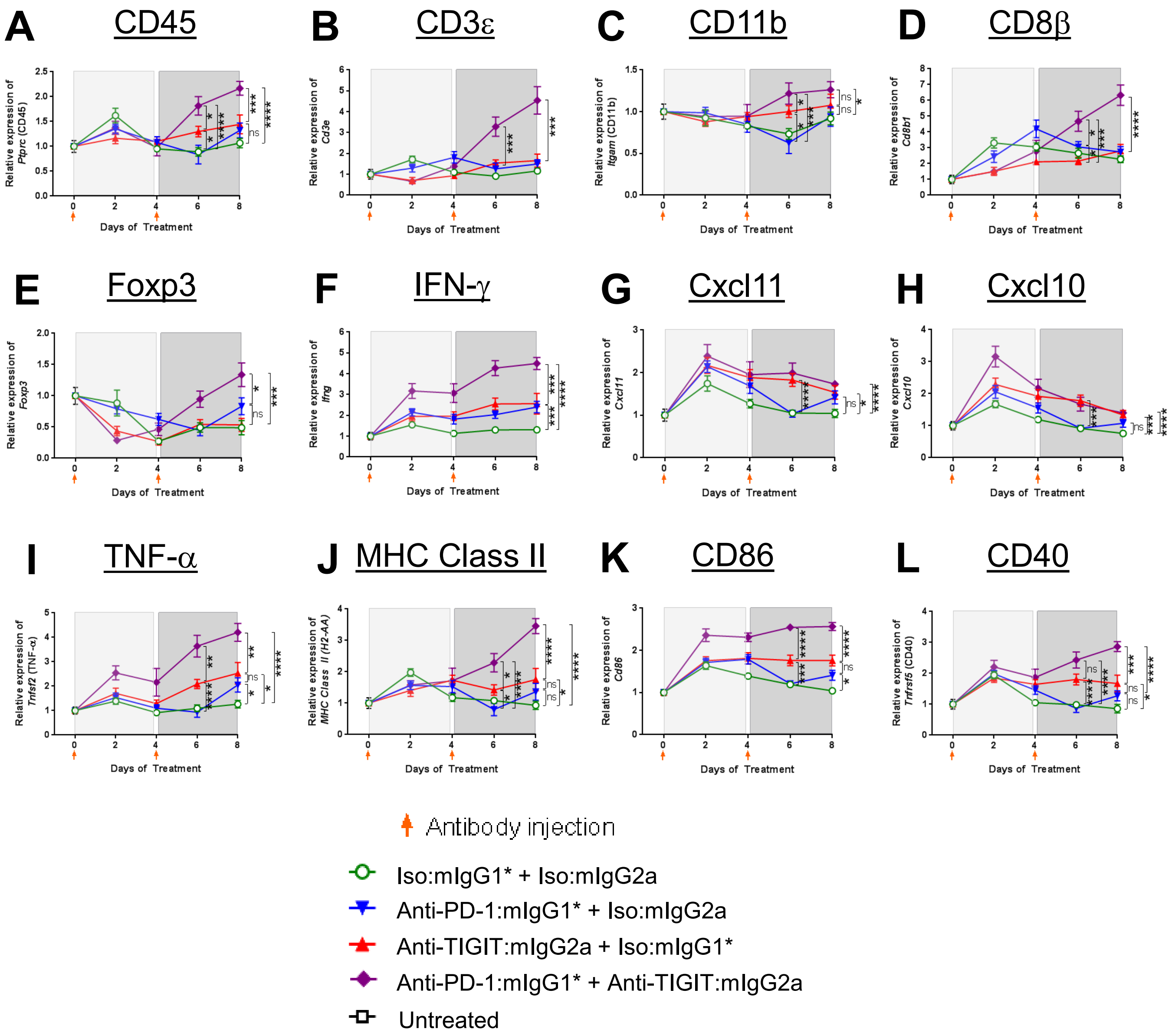

Supplementary Figure 7. Differential kinetics of relative intratumoral gene expression by mono- and combination treatments of anti-PD-1 and anti-TIGIT antibodies.

CT26 syngeneic tumor cells ($3x10^5$) were implanted subcutaneously on WT BALB/c mice. When the volume of tumor reached 116 mm^3 on average, isotype controls (Iso:mIgG1* (10 mpk) or Iso:mIgG2a (18 mpk)), anti-PD-1:mIgG1* (10 mpk) or/and anti-TIGIT:mIgG2a (18 mpk) antibodies were injected intraperitoneally every four days as indicated with orange arrows. Ten CT26 whole tumors for each group at each time point were isolated, processed, and RNA was purified for real-time PCR without any treatments ("Untreated") or after indicated treatments at day 2, 4, 6 and 8. Each data set was normalized by relative expression of *Ubb*, and the normalized values were re-calibrated to get the fold changes against the "Untreated" group. ns, not significant; *, $p < 0.05$; **, $p < 0.01$; ***, $p < 0.005$; ****, $p < 0.001$.
